# Genome size evolution in pufferfish: an insight from BAC clone-based *Diodon holocanthus *genome sequencing

**DOI:** 10.1186/1471-2164-11-396

**Published:** 2010-06-23

**Authors:** Baocheng Guo, Ming Zou, Xiaoni Gan, Shunping He

**Affiliations:** 1 Fish Phylogenetics and Biogeography Group, Key Laboratory of Aquatic Biodiversity and Conservation, Institute of Hydrobiology, Chinese Academy of Sciences, Wuhan 430072, China; 2 Graduate University of the Chinese Academy of Sciences, Beijing 100039, China; 3 Wuhan Institute of Virology, Chinese Academy of Sciences, Wuhan 430071, China

## Abstract

**Background:**

Variations in genome size within and between species have been observed since the 1950 s in diverse taxonomic groups. Serving as model organisms, smooth pufferfish possess the smallest vertebrate genomes. Interestingly, spiny pufferfish from its sister family have genome twice as large as smooth pufferfish. Therefore, comparative genomic analysis between smooth pufferfish and spiny pufferfish is useful for our understanding of genome size evolution in pufferfish.

**Results:**

Ten BAC clones of a spiny pufferfish *Diodon holocanthus *were randomly selected and shotgun sequenced. In total, 776 kb of non-redundant sequences without gap representing 0.1% of the *D. holocanthus *genome were identified, and 77 distinct genes were predicted. In the sequenced *D. holocanthus *genome, 364 kb is homologous with 265 kb of the *Takifugu rubripes *genome, and 223 kb is homologous with 148 kb of the *Tetraodon nigroviridis *genome. The repetitive DNA accounts for 8% of the sequenced *D. holocanthus *genome, which is higher than that in the *T. rubripes *genome (6.89%) and that in the *Te. nigroviridis *genome (4.66%). In the repetitive DNA, 76% is retroelements which account for 6% of the sequenced *D. holocanthus *genome and belong to known families of transposable elements. More than half of retroelements were distributed within genes. In the non-homologous regions, repeat element proportion in *D. holocanthus *genome increased to 10.6% compared with *T. rubripes *and increased to 9.19% compared with *Te. nigroviridis*. A comparison of 10 well-defined orthologous genes showed that the average intron size (566 bp) in *D. holocanthus *genome is significantly longer than that in the smooth pufferfish genome (435 bp).

**Conclusion:**

Compared with the smooth pufferfish, *D. holocanthus *has a low gene density and repeat elements rich genome. Genome size variation between *D. holocanthus *and the smooth pufferfish exhibits as length variation between homologous region and different accumulation of non-homologous sequences. The length difference of intron is consistent with the genome size variation between *D. holocanthus *and the smooth pufferfish. Different transposable element accumulation is responsible for genome size variation between *D. holocanthus *and the smooth pufferfish.

## Background

Genome size, defined as the total amount of DNA contained within the haploid chromosome set of an organism, is not only one of the most fundamental genetic properties of living organisms, but also is one of the most mysterious biological traits. A well-known phenomenon is the "C-value paradox" [1] or "G-value/N-value paradox" [2,3], which is the long-recognized lack of correlation between genome size and organism complexity [4]. A case in point is that some amoebas possess 200 times more DNA than humans [1]. Another striking characteristic of the genome size is that it can vary greatly among different taxonomic categories and even among closely related species. Genome size varies 250-fold in arthropods, 350-fold in fish, 1,000-fold in angiosperms, 5,000-fold in algae, and 5,800-fold in protozoans, and more than 200,000-fold in eukaryotes as reviewed by Gregory [5]. Genome size variation among closely related species is also prevalent and significant. For example, the fly genus *Drosophila *displays a twofold variation in genome size [6], whereas variation of genome size can achieve ninefold in a plant genus *Crepsis *[7].

Genome size variation appears to have resulted from a number of different processes over evolution time. In the short run, genome doubling or large-scale sequence duplication might be one of the most straightforward mechanisms underlying genome size variation [1,8]. The gain and loss of non-coding DNA are considered to be the main force behind the gradual accumulation of genome size variation over evolution time. For example, the correlation between genome size and transposable element amplification [9,10], and intron size variation [11-14], and gene duplication and pseudogenization [15] have been widely studied and verified across a broad phylogenetic range. Study has revealed that a massive loss of ancestral protein-coding genes has contributed to the reduction in the size of the chicken genome [16]. Overall, genome size variation is now recognized as reflecting the net effects of a collection of mechanisms that sometimes work antagonistically to expand and contract the genome, and is the result of that many forces affect collectively and operate heterogeneously among genomic regions [17,18].

**Figure 1:**
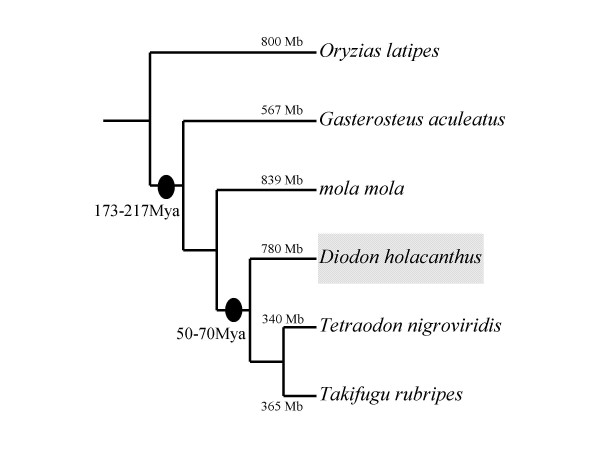
**The evolutionary history of the pufferfish**. The tree topology was modified according to Li et al. [52], and the divergent times were adopted from Steinke et al. [53] and Tyler and Santini [54]. The genome sizes of the species used in analysis are shown.

The comparative genomic analysis within closely related taxonomic groups is a powerful tool to uncover mechanisms of genome size variation, as studies in cotton [17-21] and *Drosophila *[6,22]. In this context, the pufferfish may be suitable organisms with which to study the genome size variation of vertebrates for the following reasons. The variation of genome size between tetraodontid and diodontid pufferfish presumably has resulted from a reduction of genome size in the smooth tetraodontid pufferfish relative to their spiny diodontid relatives since their divergence during the 50-70 million years ago (Figure 1) [23]. In the family Tetraodontidae, the smooth pufferfish have the smallest vertebrate genomes known to date, which are one-eighth the length of the human genome, with a haploid genome size 365 million bp in *T. rubripes *[13] and 340 million bp in *Te. nigroviridis *[24]. The spiny pufferfish in Diodontidae, the sister family of Tetraodontidae, have genomes that are roughly twice as large, about 800 Mb [23,25,26]. *Mola mola *(Molidae), a member of the closest outgroup to Tetraodontidae and Diodontidae, has a genome size around 800 Mb [23,26]. Therefore, the variation of genome size in pufferfish, especially the highly reduced genome size in the smooth pufferfish, makes pufferfish a good model system to study genome size shrinking in vertebrates. Furthermore, because of possessing small genomes, the smooth pufferfish (*T. rubripes *and *Te. nigroviridis*) have been used as model organisms and their genomes have been sequenced [13,24]. It is feasible to investigate the dynamics of genome size variation in large-scale genomic region rather than in particular genome region [17,18].

By combining the results of the DNA renaturation kinetics analysis and rates of small (< 400 bp) insertions and deletions, Neafsey and Palumbi [27] proposed that the unequal rates of large insertions have contributed to the genome size variation between tetraodontid and diodontid pufferfish since their divergence, and genome compacting in the tetraodontid lineage is attributable to a reduction in the rate of large insertions rather than to an increase in large deletions. Aparicio et al [13] demonstrated that the genome of *T. rubripes *is compact partly because its introns are shorter than those of the human genome. To better understand the variation of genome size among pufferfish, it may be informative to compare closely related species within a phylogenetic framework using a comparative genomic approach by fully utilizing the whole genome sequence resources of *T. rubripes *and *Te. nigroviridis*.

Therefore, in this study, we have examined the genome of the spiny pufferfish *D. holocanthus *by sequencing bacterial artificial chromosomes (BAC) and conducted comparative genomic analyses with the genomes of the smooth pufferfish *T. rubripes *and *Te. nigroviridis*. The possible forces related to genome size variation were analyzed. Synteny analysis showed that less 50% of the sequenced *D. holocanthus *genomic sequences could be aligned with the genomes of tetraodontid pufferfish *T. rubripes *and *Te. nigroviridis*, which is consistent with their different genome size level. The repeat element component of the sequenced *D. holocanthus *genome was 7.94%, which is higher than the total repeat element component of whole genomes of *T. rubripes *(6.89%) and *Te. nigroviridis *(4.66%). The differences of repeat element components in both the homologous regions and the non-homologous regions between *D. holocanthus *and tetraodontid pufferfish are large and significant, especially the proportion of retroelements. The differences of intron size between *D. holocanthus *and the smooth pufferfish are also significant. Our results showed that intron size variation was consistent with genome size variation between *D. holocanthus *and smooth pufferfish. We verified that different amount and content of transposable elements were responsible for the genome size variation between *D. holocanthus *and smooth pufferfish.

## Results

### Sequence assembly and annotation

A BAC library of the spiny pufferfish *D. holocanthus *was constructed and used for BAC clone selection in this study. Ten clones, containing sequences of around 100 kb, were randomly selected and subsequently sequenced at the Beijing Genomics Institute. A total of 9,286 high-quality reads produced a 2.84 Mb data set. By combining with the estimated length of each BAC clone, we determined that the average coverage for draft sequences of these BAC clones is approximately 3.14-fold. Further assembly generated 49 scaffolds, ranging from 2.2 kb to 66.6 kb and representing a total of 776 kb of non-redundant sequences without gaps and 822 kb of non-redundant sequences with gaps of the *D. holocanthus *genome. The detailed sequencing information is given in Table 1.

**Table 1:** Sequencing information of each BAC clone.

*BAC ID*	*Read numbers*	*Read lengths(bp)*	*Assembled* *length(bp)*	*Estimated size (kb)*	*Coverage*	*N50 (bp)*
ctfa	1020	311030	87525	90	3.46	78386
ctfb	1013	335538	103323	110	3.05	67071
ctfc	1000	324833	99742	95	3.42	51904
ctfd	996	303386	89282	90	3.37	54060
ctfe	890	279782	80143	100	2.80	41374
ctff	881	251821	75743	80	3.15	40883
ctfg	794	255225	62595	90	2.84	44454
ctfh	942	281266	85394	80	3.52	66638
ctfj	655	186358	62614	70	2.66	39606
ctfk	1095	315029	85969	100	3.15	43834
Total	9286	2844268	832330		3.14	

Coverage, regarded as sequencing redundancy was calculated as the ratio of the total read length to the assembled length. N50 length is the size x such that 50% of the assembly is in units of length at least x.

The GC content of the sequenced *D. holocanthus *genome was 41.65%. Gene annotation of these sequences predicted 87 putative genes, including alternatively splicing transcript forms. In the predicted gene set, 62 genes had complete open reading frames, ranging from 204 bp to 13.8 kb with an average length of 1517 bp and 8 coding exons per gene on average. In the predicted gene set, totally 619 complete exons existed and had an average length of 194 bp and 532 introns had an average length of 845 bp. The coding regions accounted for approximately 120 kb, or 15.5% of the sequenced *D. holocanthus *genome in total. In the predicted gene set, 27 of the predicted genes that were predicted by both FgenesH 2.6 [28] and GENSCAN 1.0 [29] had no significant hits when searched against the non-redundant GenBank protein database with BLASTP [30]. This subset of genes was named as novel predicted proteins. Names of remaining gene-encoded proteins were assigned according to the BLASTP searches. Possible alternatively spliced forms of 10 genes were annotated in the predicted gene set. Finally, a total of 77 distinct genes with either complete or incomplete coding sequences (CDS) were found in the sequenced BAC clones dataset. Detailed information regarding the predicted gene set of the spiny pufferfish is given in Table 2.

**Table 2:** Summary of the predicted genes in each BAC clone.

*BAC ID*	*Scaffold ID*	***GenBank Acc. No***.	*Gene Name*
ctfa	Scaffold000001	GU002104	ring finger protein 17;centromere protein J; similar to transcription termination factor, RNA polymerase II
	Scaffold000002	GU002105	hypothetical protein LOC334519; unnamed protein product
	Scaffold000003	GU002106	similar to ReO_6; similar to ring finger protein 17
ctfb	Scaffold000001	GU002107	similar to GF20795; similar to KH domain-containing, RNA-binding, signal transduction-associated protein 2; EGF-like domain-containing protein; novel protein similar to vertebrate PHDfinger protein 3 (PHF3)
	Scaffold000002	GU002108	
	Scaffold000003	GU002109	similar to KH domain-containing, RNA-binding, signal transduction-associated protein 2
	Scaffold000004	GU002110	similar to DNA primase large subunit(58 kDa)
	Scaffold000005	GU002111	
ctfc	Scaffold000001	GU002112	novel predicted protein; similar to neuron navigator 1; similar to cysteine and glycine-rich protein 2
	Scaffold000002	GU002113	similar to cardiac troponin T; similar to plakophilin 1
	Scaffold000003	GU002114	similar to TEA domain family member 3
	Scaffold000004	GU002115	similar to troponin I, slow skeletal muscle
ctfd	Scaffold000001	GU002116	similar to neurocan; novel predicted protein
	Scaffold000002	GU002117	unnamed protein product; novel predicted protein; similar to hypothetical LOC100002099; similar to deoxyhypusine hydroxylase/monooxygenase; transmembrane 6 superfamily member 1-like
	Scaffold000003	GU002118	similar to phosphodiesterase 4D-interacting protein; unnamed protein product
	Scaffold000004	GU002119	
	Scaffold000005	GU002120	unnamed protein product
ctfe	Scaffold000001	GU002121	glutathione S-transferase theta; novel predicted protein
	Scaffold000002	GU002122	novel predicted protein; d-dopachrome decarboxylase; similar to coiled-coil-helix-coiled-coil-helix domain containing 10; zinc finger 214-contain protein
	Scaffold000003	GU002123	
ctff	Scaffold000001	GU002124	similar to human adrenergic receptor kinase 2; similar to human adrenergic receptor kinase 2
	Scaffold000002	GU002125	similar to beta-adrenergic kinase 2; novel predicted protein
	Scaffold000003	GU002126	similar to myosin-like protein
	Scaffold000004	GU002127	similar to beta-adrenergic receptor kinase 2
	Scaffold000005	GU002128	similar to beta-adrenergic receptor kinase 2
	Scaffold000006	GU002129	
	Scaffold000007	GU002130	
	Scaffold000008	GU002131	claudin 5b
	Scaffold000009	GU002132	novel predicted protein
ctfg	Scaffold000001	GU002133	novel predicted protein; similar to tetraspanin 18; novel predicted protein
	Scaffold000002	GU002134	similar to erythrocyte membrane protein 3; novel predicted protein
	Scaffold000003	GU002135	aristaless-like homeobox 4
ctfh	Scaffold000001	GU002136	novel predicted protein; similar to myocyte enhancer factor 2a; novel predicted protein; similar to multiple C2 and transmembrane domain-containing protein 2
	Scaffold000002	GU002137	novel predicted protein; novel predicted protein; novel predicted protein
	Scaffold000003	GU002138	
ctfj	Scaffold000001	GU002139	novel predicted protein; novel predicted protein; similar to sterile alpha motif domain containing 3; similar to zinc finger protein
	Scaffold000002	GU002140	novel predicted protein; novel predicted protein
	Scaffold000003	GU002141	unnamed protein product
	Scaffold000004	GU002142	Dexamethasone-induced Ras-related protein 1 precursor
	Scaffold000005	GU002143	
	Scaffold000006	GU002144	
ctfk	Scaffold000001	GU002145	similar to calcium-transporting ATPase 2C1 isoform 1a; similar to aldehyde dehydrogenase 5 family, member A1; similar to KIAA0319; Similar to DENN/MADD domain-containing 4B
	Scaffold000002	GU002146	novel predicted protein; similar to calcium-transporting ATPase 2C1
	Scaffold000003	GU002147	novel predicted protein
	Scaffold000004	GU002148	
	Scaffold000005	GU002149	
	Scaffold000006	GU002150	
	Scaffold000007	GU002151	novel predicted protein
	Scaffold000008	GU002152	

Novel predicted proteins represent predicted genes that had no significant hits when searched against the non-redundant GenBank protein database using BLASTP. Genes used for the comparisons of intron size variation are underlined.

### Comparative mapping and synteny identification

**Figure 2:**
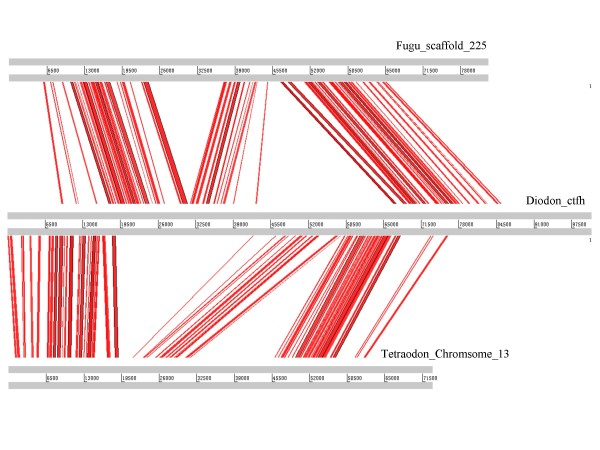
**Microsynteny between the sequences of clone ctfh in *D. holocanthus *genome and its homologous region in *T. rubripes *and *Te. nigroviridis *genome**. The length of the bar represents the relatively length of the sequence, and the lines between the bars represent the high-scoring segment pair (HSP) of the BLASTN searches between the sequences. Other microsyntenic relationships between the BAC clone sequences of *D. holocanthus *and the smooth pufferfish genomes were given in additional file 1.

To identify homologous regions and sequence similarity patterns between the sequenced genome of *D. holocanthus *and the genomes of other species, we performed BLASTN searches against genomes of *T. rubripes*, *Te. nigroviridis*, *Gasterosteus aculeatus*, and *Oryzias latipes*. The genomic synteny regions between the sequenced genome of *D. holocanthus *and the genomes of other model fish were listed in Table 3. The *T. rubripes *genome had the highest sequence similarity to the sequenced *D. holocanthus *genome, and 46.9% of the sequenced BAC sequences could be aligned with the genome of *T. rubripes*, 28.8% with the *Te. nigroviridis *genome, 22.1% with the *G. aculeatus *genome, and 20.9% with the *O. latipes *genome. Based on the results of the BLASTN searches (see Materials and Methods), 30 scaffolds of the sequenced BAC clones had syntenic regions and were localized on 12 scaffolds in *T. rubripes*, and the total length of the homologous regions was 364 kb in *D. holocanthus *and was 265 kb in *T. rubripes*; 25 BAC-sequenced scaffolds were localized on chromosome 2, 12, 13, and 17, and Un_random region in *Te. nigroviridis*, and the total length of the homologous regions was 223 kb in *D. holocanthus *and was 148 kb in *Te. nigroviridis*; 26 BAC-sequenced scaffolds were localized on 10 groups and 2 scaffolds in *G. aculeatus*, and the total length of the homologous regions was 171 kb in *D. holocanthus *and was 168 kb in *G. aculeatus*; 24 BAC-sequenced scaffolds were localized on chromosome 1, 5, 6, 12, 15, and 17, and scaffold 1954 and 4255 in *O. latipes*, and the total length of the homologous regions was 162 kb in *D. holocanthus *and was 159 kb in *O. latipes*. Figure 2 shows the synteny relationships of scaffolds of clone ctfh in the spiny pufferfish with *T. rubripes *and *Te. nigroviridis*. Fifteen BAC-sequenced scaffolds of the sequenced *D. holocanthus *genome were sequence-conserved and could be localized on all genomes of the other four model fish species. Eleven BAC-sequenced scaffolds, representing 100 kb or 12.1% of the sequenced *D. holocanthus *genome, had no homologous regions in the other four fish genomes. Fourteen BAC-sequenced scaffolds, representing a total length of 100 kb sequences or 12.2% of the sequenced *D. holocanthus *genome, could not be localized on the genomes of *T. rubripes *and *Te. nigroviridis*, but two of these sequences had homologous regions in *G. aculeatus *or *O. latipes*. Ten BAC-sequenced scaffolds displayed homologous relationships to the specific regions of the *T. rubripes *genome but had no synteny regions in the genome of *Te. nigroviridis*, whereas five BAC-sequenced scaffolds can be localized on the genome of *Te. nigroviridis *but had no synteny regions in the genome of *T. rubripes*.

**Table 3:** Locations of each BAC on the genomes of different species

*BAC ID*	*Scaffold*	*T.rubripes*	*Te. nigroviridis*	*G. aculeatus*	*O. latipes*
ctfa	Scaffold000001	scaffold_38	Chromosome_2	scaffold_115	Chromosome_21
	Scaffold000002	scaffold_38		groupXIV	
	Scaffold000003				
ctfb	Scaffold000001	scaffold_24	Chromosome_17	groupVI	Chromosome_15
	Scaffold000002	scaffold_24	Chromosome_17	groupXI	
	Scaffold000003	scaffold_24	Chromosome_17	groupVI	Chromosome_15
	Scaffold000004	scaffold_24	Chromosome_17	groupVI	Chromosome_15
	Scaffold000005				scaffold3867
ctfc	Scaffold000001	scaffold_635	Un_random	groupXVII	Chromosome_5
	Scaffold000002	scaffold_47	Un_random	groupXVII	
	Scaffold000003	scaffold_47	Un_random	groupXVII	Chromosome_5
	Scaffold000004			groupXVII	Chromosome_5
ctfd	Scaffold000001	scaffold_200		groupIII	Chromosome_17
	Scaffold000002	scaffold_200		groupIII	Chromosome_17
	Scaffold000003	scaffold_200		groupIII	Chromosome_17
	Scaffold000004	scaffold_200	Un_random	groupIII	Chromosome_17
	Scaffold000005	scaffold_200		groupIII	
ctfe	Scaffold000001				
	Scaffold000002	scaffold_589	Un_random		
	Scaffold000003				
ctff	Scaffold000001	scaffold_27			Chromosome_12
	Scaffold000002	scaffold_27			
	Scaffold000003	scaffold_27	Un_random		Chromosome_12
	Scaffold000004	scaffold_27			
	Scaffold000005	scaffold_27			
	Scaffold000006	scaffold_27			Chromosome_12
	Scaffold000007				
	Scaffold000008	scaffold_50	Chromosome_12		
	Scaffold000009				
ctfg	Scaffold000001	scaffold_2	Chromosome_13	groupXIX	Chromosome_6
	Scaffold000002				
	Scaffold000003	scaffold_2	Chromosome_13	groupXIX	Chromosome_6
ctfh	Scaffold000001	scaffold_225	Chromosome_13	groupXIX	Chromosome_6
	Scaffold000002	scaffold_225	Chromosome_13	groupXIX	Chromosome_6
	Scaffold000003	scaffold_225	Chromosome_13	groupXIX	Chromosome_6
ctfj	Scaffold000001		Un_random	groupXIII	Chromosome_1
	Scaffold000002		Chromosome_2	groupXIII	
	Scaffold000003		Un_random		
	Scaffold000004	scaffold_3	Chromosome_2	groupV	scaffold4255
	Scaffold000005		Chromosome_2		
	Scaffold000006				
ctfk	Scaffold000001	scaffold_270	Un_random	groupXX	scaffold1954
	Scaffold000002	scaffold_270	Un_random	groupXX	scaffold1954
	Scaffold000003		Chromosome_2		
	Scaffold000004				
	Scaffold000005				
	Scaffold000006				
	Scaffold000007				
	Scaffold000008			scaffold_56	

### Intron size variation

To investigate size intron variation between *D. holocanthus *and the smooth pufferfish, pairwise comparisons were performed. The predicted gene dataset of the sequenced *D. holocanthus *genome contained a total of 692 complete exons with an average length 208 bp and 621 introns with an average length 868 bp. To accurately estimate size variations of intron of these species, the structures of the predicted genes with complete open reading frames were re-determined using GeneWise [31], a widely used tool that exquisitely predicted gene structure based on similarity searches against proteins. Finally, 10 genes (see Table 2) with high reliability (GeneWise score > 100) were selected from the predicated gene dataset for intron size comparisons. After the introns with sequence gaps were excluded, a total of 125 gap-free introns were used for comparison with their orthologues in the other model fish species. Orthologous genes were retrieved directly from the Ensembl ortholog prediction dataset (release 52) in *T. rubripes*, *Te. nigroviridis*, and *G. aculeatus*.

**Figure 3:**
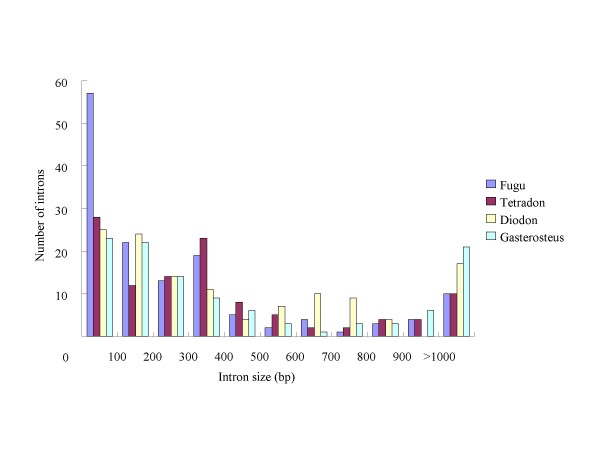
**Distribution patterns of intron size**. Distributions of the intron lengths used for the comparison in *D. holocanthus *(Diodon), *T. rubripes *(Fugu), *Te. nigroviridis *(Tetraodon), and *G. aculeatus *(Gasterosteus).

The length distribution patterns of intron size are shown in Figure 3. The numbers and mean lengths of the intron size are listed in Table 4. The modal values of intron size appear in the range of 1-100 bp in all of these four species, with 83% of introns in *T. rubripes *and 76% of introns in *Te. nigroviridis *< 500 bp, whereas only 62% of introns in *D. holocanthus *and 66% of introns in *G. aculeatus *are shorter than 500 bp. The proportion of intron longer than 1000 bp was 7.1% in *T. rubripes*, 8.9% in *Te. nigroviridis*, 13.6% in *D. holocanthus*, and 18.9% in *G. aculeatus*. The mean length of intron in the *T. rubripes *genome was 435.2 bp and was almost equal to that (434.9 bp) in the *Te. nigroviridis *genome. The mean intron length in *D. holocanthus *is 566 bp which is obviously greater than that in the smooth pufferfish. The mean intron length in three-spined stickleback is clearly greater than that in the pufferfish. Because of the non-normal distribution of intron size (Kolmogorov-Smirnov normality test, *p *= 4.82e-007), the Wilcoxon signed rank test, a non-parametric test, was adopted and detected a significant differences in the intron lengths between *D. holocanthus *and *T. rubripes *(*p *= 2.96e-022), between *D. holocanthus *and *Te. nigroviridis *(*P *= 5.30e-003), between *D. holocanthus *and *G. aculeatus *(*P *= 2.52e-013), between *G. aculeatus *and *T. rubripes *(*P *= 1.31e-019), and between *G. aculeatus *and *Te. nigroviridis *(*P *= 1.56e-006).

**Table 4:** Comparison of the average intron size and exon lengths of *D. holocanthus*, *T. rubripes*, and *Te. nigroviridis*.

	*D. holocanthus*	*T. rubripes*	*Te. nigroviridis*	*G. aculeatus*
**Intron number**	125	140	111	110
**Average intron length (bp)**	566	435	435	722

### Repetitive elements in pufferfish genome

The different accumulation of repeat elements is recognized as a prominent force in genome size variation. Therefore, we examined the sequenced BAC clones and the smooth pufferfish genomes for evidence of repeat elements (Table 5). In total, 431 tracts of repeat element were detected in the sequenced spiny pufferfish genome. The total length of repeat elements was approximately 62 kb and accounted for 7.94% of the sequenced BAC clones. No small RNA or satellite repeats were detected. Simple repeats and low complexity repeats accounted for 0.75% and 0.46% of the sequenced spiny pufferfish genome, respectively. Our analysis result showed 6.73% of the sequenced BAC clones to match interspersed repeats. A large fraction of the interspersed repeats was contributed by retroelements (6.00% of the BAC clone sequences), whereas 0.66% comprised DNA transposons and 0.07% unclassified interspersed repeats. We catalogued the transposable elements in the sequenced BAC clones in detail, and identified 21 subfamilies of transposable elements belonging to 12 known families (Table 6). The long interspersed repetitive elements (LINEs) constituted more than half the transposable elements and 3.44% of the BAC sequences. Four LINE families, LINE1, LINE2, Rex1/Babar, and RTE, were detected, with 29 copies Expander representing the RTE family and 13 copies Maui belonging to the L2 family. Two short interspersed repetitive element (SINE) families V-SINE (81 copies) and Mermaid (25 copies), were found in the BAC clone sequences. One long terminal repeat (LTR) retrotransposons family, Gypsy/TY3 (4 copies), were identified. Four DNA transposon families, hAT-Charlie, Harbinger, Tc1-mariner, and Tc2, were detected and accounted for 0.66% of the BAC clone sequences. Our analysis showed that most of the repeat elements were distributed within genes (e.g., 67 of 106 copies of SINEs fall into introns, as do 77 of 135 copies of simple repeats) in the sequenced region of the *D. holocanthus *genome.

**Table 5:** Comparison of repetitive DNA sequence contents (%) of *D. holocanthus*, *T. rubripes*, and *Te. nigroviridis*

*Component*	*D. holocanthus*			*T. rubripes*		*Te. nigroviridis*	
	
	WG	HR-fugu	HR-tetra	WG	HR-Dh	WG	HR-Dh
**Bases masked (%)**	7.94	5.16	4.87	6.89	2.43	4.66	2.93

**Interspersed repeats**	6.73	4.05	3.84	4.69	1.03	2.03	0.18
**Retroelements**	6.00	3.76	3.36	3.39	0.33	1.37	0.08
**SINEs**	2.00	1.63	1.15	0.20	0.00	0.13	0.00
**LINEs**	3.44	2.13	2.12	2.51	0.22	1.06	0.08
**LTR elements**	0.55	0.00	0.00	0.68	0.11	0.19	0.00
**DNA transposons**	0.66	0.29	0.48	1.05	0.61	0.45	0.00
**Rolling-circles**	0.00	0.00	0.00	0.00	0.00	0.00	0.00
**Unclassified**	0.07	0.00	0.00	0.25	0.09	0.21	0.10
**Small RNA**	0.00	0.00	0.00	0.00	0.00	0.00	0.00
**Satellites**	0.00	0.00	0.00	0.01	0.00	0.00	0.00
**Simple repeats**	0.75	0.68	0.75	1.75	0.93	1.96	2.34
**Low complexity**	0.46	0.43	0.28	0.44	0.47	0.67	0.41

WG, sequenced whole genome dataset; HR-fugu, homologous region in the *D. holocanthus *genome compared with the *T. rubripes *genome; HR-tetra, homologous region in the *D. holocanthus *genome compared with the *Te. nigroviridis *genome; HR-Dh, homologous regions in the smooth pufferfish genomes compared with the *D. holocanthus *genome.

**Table 6:** Transposable elements in *D. holocanthus *and their classification

*Repeat classification*	*Distribution*	*Fugu members*	*Copy number*
SINEs			
V	Vertebrates	FR2	63 (46)
		TE	18 (11)
Mermaid	Vertebrates	FR1c	16 (5)
		FR1d	9 (3)
Non-LTR Retrotransposons			
L1	Vertebrates, plants	KibiFr1	2
RTE	Nematodes	Expander	29
		Expander2	2
L2	Metazoa	Maui	13
Rex1/Babar	Fish	Rex1_FurC	7
LTR Retrotransposons			
Gypsy/TY3	Eukaryotes	Ronin1_I	4
		Ronin2_I	1
		Ronin3_I	1
		Samurai_I	1
Penelope-like	Insects, fish	Bridge2(Xena)	17
DNA transposons			
hAT-Charlie	Mammals	Chaplin4_FR	2 (2)
Harbinger	Nematodes, plants	Senkusha1A	2 (1)
Tc1-mariner	Metazoa, plants	TC1_FR1	8 (3)
		TC1_FR3	2 (0)
		TC1_FR4	5 (3)
Tc2	Nematodes	TC2_FR2a	2 (2)
		TC2_FR4	1 (1)
Unclassified			6 (1)

Numbers in parentheses represent the number of transposable elements distributed within gene.

To accurately estimate the contribution of repeat elements to pufferfish genome size variation, we compared the proportion of repeat elements in the homologous regions between the spiny pufferfish *D. holocanthus *and the smooth pufferfish based on the synteny analysis (Table 5). In the homologous regions between *D. holocanthus *and *T. rubripes*, repeat elements accounted for approximately 19 kb and 5.16% of the sequences in *D. holocanthus*, whereas the total length of the repeat elements was 6,457 bp, accounting for 2.43% of the homologous regions in *T. rubripes*. In the homologous regions between *D. holocanthus *and *Te. nigroviridis*, repeat elements accounted for approximately 11 kb and 4.87% of the sequences in *D. holocanthus*, whereas accounted 4,304 bp or 2.93% of homologous sequences in *Te. nigroviridis*. No small RNA or satellite repeats were detected in their homologous regions. Although their sequence lengths were almost equal, the proportion of low complexity repeats in *D. holocanthus *was lower than those in the smooth pufferfish. The proportions of simple repeats in the smooth pufferfish were much higher than that in *D. holocanthus*. Unlike *D. holocanthus*, simple repeat elements contributed the major fraction (almost 80%) of the repeat elements in homologous region of *Te. nigroviridis*. The proportion of simple repeats was 2.34% in the *Te. nigroviridis*, and was more than three times larger than it (0.75%) in *D. holocanthus*. The total length of simple repeats was 3,435 bp in *Te. nigroviridis *and was 1,666 bp in *D. holocanthus*.

In the homologous regions between *D. holocanthus *and *T. rubripes*, the total length of interspersed repeats was approximately 15 kb and accounted for 4.05% of the homologous sequences in *D. holocanthus*, whereas in *T. rubripes *the length was 2,748 bp and accounted for 1.03% of the homologous regions. SINEs were absent from *T. rubripes*, but 39 copies of SINEs, including 30 copies of V-SINE and 9 copies of SINE/Mermaid, were identified in *D. holocanthus*. The total length of SINEs in *D. holocanthus *was 5,944 bp, accounting for 1.63% of the homologous regions. The proportion of the LINEs in *D. holocanthus*is 2.13%, which is almost 10 times higher than it (0.22%) in *T. rubripes*. In *D. holocanthus *21 partial copies of LINEs were detected, whereas there were only 3 partial copies in *T. rubripes*. No LTR retrotransposon was detected in *D. holocanthus*, whereas 2 copies were identified in *T. rubripes*. Unlike retroelements, the proportion of DNA transposons in *T. rubripes *was more than twice as high as that in *D. holocanthus*. The proportions of the interspersed repeats varied significantly in the homologous regions between *D. holocanthus *and *Te. nigroviridis*. In *D. holocanthus*, the proportion was 3.84% (8573 bp), with 1.15% of SINEs, 2.12% of LINEs, and 0.48% DNA transposons. In *Te. nigroviridis*, the proportion is only 0.18%, with 0.08% of LINEs and 0.10% of unclassified interspersed repeat, whereas SINEs are absent. Similar to the distribution pattern in the sequenced *D. holocanthus *genome, most of the repeat elements in *D. holocanthus *fell within genes in the homologous regions shared with the smooth pufferfish, e.g., 27 out of 37 SINE copies were integrated within introns in its homologous regions shared with *T. rubripes *and 8 out of 17 copies occurred in the homologous region it shares with *Te. nigroviridis*.

## Discussion

### Character of the *D. holocanthus *genome

In recent years, the use of comparative genomic approach to study genome size variation within a phylogenetic framework has shed much light on the genome size variation in closely related species, such as studies in cotton [17,18,20,21,32], in rice [10], and in *Drosophila *[6,22]. Here, to study the genome size variation in pufferfish, especially the genome shrinking of the smooth pufferfish, a total length of 776 kb of non-redundant sequences or 0.1% of the spiny pufferfish *D. holocanthus *genome (780 Mb) was sequenced and compared with the smooth pufferfish genomes. The GC level of the sequenced *D. holocanthus *genome (41.65%) is within the vertebrate range, between 40% for *Bos taurus *and 48% for *Sus scrofa *[33], but it is lower than it in the genome of *T. rubripes *(45.46%) and *Te. nigroviridis *(46.43%), whereas it is close to it in *O. latipes *genome (40.46%). Because GC content represents gene density to some extent, the spiny pufferfish should have a lower gene density genome compared with the smooth pufferfish with compact genomes. This pattern is also supported by the proportion of coding region in the sequenced *D. holocanthus *genome. The coding region accounted for 15.5% of the assembled spiny pufferfish genome, whereas in *Te. nigroviridis *it accounted for 40% of the genome [24]. Our gene annotation results showed that the mean number of coding exons per gene with complete CDS on average (8 when untranslated regions are excluded) in the *D. holocanthus *genome is close to that in human genome (8.7) and mouse genome (8.4) [34], but is larger than that in the *Te. nigroviridis *(6.9) genome [24]. Assuming that fish and mammal genes have similar structures, this suggests that our gene prediction results are trustworthy and that some annotated genes of *Te. nigroviridis *are partial or fragmental as suggested by Jaillon et al. (2004) [24]. Our repeat element analysis showed that the sequenced *D. holocanthus *genome contains more repeat elements, especially the interspersed elements, than do the smooth pufferfish genomes, which is consistent with the DNA renaturation analysis in previous study [27]. Thus, *D. holocanthus *has a lower gene density and repeat elements rich genome compared with smooth pufferfish. It is noteworthy that no inconsistency for the arrangement of scaffolds was detected during comparison with the smooth pufferfish genomes, which suggests that the order of assembled scaffolds is reliable and implies that negligible genomic rearrangement has occurred in the sequenced region of the *D. holocanthus *genome at the scale of the BAC clones (~100 kb).

### Genome size evolution in pufferfish

Variations in genome size within and between species have been observed since the 1950 s in diverse taxonomic groups. Interestingly, the smooth pufferfish have the smallest vertebrate genomes known to date, and evolved toward a high level of compact organization. Within the pufferfish, the genome of *D. holocanthus *is almost twofold larger than that of the smooth pufferfish. However, the difference of genome size between this spiny puffer and the smooth puffer does not appear to reflect a difference in ploidy [23], although the karyotype of *D. holocanthus *is unknown. The chromosome number (2n) ranges from 34 to 44 in the smooth pufferfish, and one spiny pufferfish *D. bleekeri *karyotyped to date has 46 chromosomes [35]. Parsimony analysis of the phylogenetic patterns of genome size in pufferfish suggests that the smooth pufferfish and the spiny pufferfish should have a plesiomorphic genome size of 800-900 Mb and that the tiny genome of the smooth pufferfish is a derived character and unique to the smooth pufferfish [23]. Our synteny analysis supports this conclusion to some extent, insofar as doubled regions homologous of the smooth pufferfish genome were not detected in the sequenced *D. holocanthus *genome and less than half the BAC clone sequences can be aligned with the smooth pufferfish genomes. Thus, the almost twofold size difference between *D. holocanthus *and the smooth pufferfish genomes must be the result of other processes, which have contributed to this genome size variation over the long evolution timescales.

Previous studies have revealed that intron size variation is positively correlated with genome size variation [11,12,22]. Compared with human, the compact genome of *T. rubripes *is suggested to correlate positively with intron size shrinking [13,36]. In our analysis, we observed a correlation between the size of genome and intron length in pufferfish, with the smooth pufferfish having both smaller genomes and shorter introns compared with *D. holocanthus *having relative larger genome size and long introns. Statistical analysis showed that the difference of the average intron length between *D. holocanthus *(mean of 566 bp) and the smooth pufferfish (mean of 435 bp) was statistically significant. Although only a tiny fraction of the introns was sampled in our analysis, the nearly identical length distribution patterns (Figure 3) compared with that in the *T. rubripes *genome [13] suggested that our sampled data were not biased and the result was robust. Additionally, *G. aculeatus *was used as outgroup for intron size comparisons. Our results showed that the intron size in the *G. aculeatus *genome was significantly larger than that in pufferfish genomes, which suggested that different the intron size variation between the smooth pufferfish and the spiny pufferfish contributed to their genome size variation since their divergence with *G. aculeatus*. According to our analysis result, the intron length variation involved 31 Mb sequence and accounts for approximately 7.21% of the genome size variation between *D. holocanthus *and smooth pufferfish, if 30,000 genes exist in pufferfish genome as suggested by Jaillon et al [24]. The correlation between the genome size and intron size in pufferfish is consistent with that observed in *Drosophila *[22], bird [11], and *Muntiacus muntjak vaginalis *[14], but differs from that in plants (e.g., *Gossypium *[21]). It seems that the correlation between intron and genome sizes depends on both the phylogenetic distance of the organisms studied and the time of their divergence. In fact, one explanation of to this phenomenon is that the rate of accumulation of insertions and deletions (indels) in intron sequence has varied in different organisms over evolution time [37,38]. For example, as one of the main insertion forces, transposable elements affect plant and mammalian genomes in different ways. Most transposable element insertions occur in the intergenic regions of plant genomes [39,40], but within intragenic regions (introns) of mammalian genomes [41]. As in the mammalian genomes, we found that most of the repeat elements in the sequenced *D. holocanthus *genome are mainly distributed in introns and have partially contributed to the difference of intron size between *D. holocanthus *and the smooth pufferfish. Another potential mechanisms responsible for the intron size variation between *D. holocanthus *and the smooth pufferfish may be the accumulation of small indels over their evolution time, as has occurred in the in cotton genus [32]. However, small indels cannot be detected in intron sequence between *D. holocanthus *and smooth pufferfish because of their relatively long divergence time (Figure 1). Thus, our comparison of the intron sizes of orthologous genes between *D. holocanthus *and the smooth pufferfish strongly supports the proposition that intron size variation resulting from different repeat element insertions and different accumulation of indels have contributed to genome size difference between *D. holocanthus *and the smooth pufferfish, which is consistent with the conclusion drawn in previous studies [11-14,22,36]. Overall, our results demonstrated that genes in the *D. holocanthus *genome are characterized by significant expansion in their intron size compared with the smooth pufferfish and accounted for genome size variation in the pufferfish. Genome size variation takes place also within genes in pufferfish.

In addition to intron size variation, the most potential force responsible to genome size variation is the differential accumulation and deletion of transposable elements, which have been observed and verified across a broad phylogenetic range of organisms including plants [20] and mammals [42,43]. A previous DNA renaturation analysis showed that middle-repetitive DNA was underrepresented in the smooth pufferfish genomes compared with a spiny pufferfish (*D*. *hystrix*) genome, and that a significantly greater abundance of a transposon-like repetitive DNA class existed in the spiny pufferfish genome relative to the smooth pufferfish genomes [27]. In our analysis, the proportion of repeat elements in the sequenced *D. holocanthus *genome (7.94%) is higher than that in the smooth pufferfish genomes, with 6.89% in the *T. rubripes *genome and 4.66% in the *Te. nigroviridis *genome. Among the repeat elements, the fraction of interspersed repeats in the *D. holocanthus *genome was much higher than that in the smooth pufferfish genomes, but the proportion of simple repeats is lower. Our result is consistent with the result of DNA renaturation analysis [27]. We estimated here that the number of the interspersed repeats in the *D. holocanthus *genome (193,000, calculated from 193 copies in the 0.1% sequenced genome region) far exceeds that in the *T. rubripes *genome (46,000 copies) and that in the *Te. nigroviridis *genome (19,000 copies). To ensure that this pattern did not result from sample bias because only part of the *D. holocanthus *genome was available for analysis, we identified the repeat elements in the homologous regions between *D. holocanthus *and the smooth pufferfish genomes. This pattern recurred with an increasing discrepancy in the interspersed repeat fraction. In the homologous regions, the proportion of interspersed repeats in the *D. holocanthus *genome was nearly four times higher than that in the *T. rubripes *genome and more than 20 times higher than that in the *Te. nigroviridis *genome (Table 5). We estimated that the different profiles of repeat element sequences accounted for 12.5% of the homologous region variation between *D. holocanthus *and *T. rubripes *and for 8.59% between *D. holocanthus *and *Te. nigroviridis*. In the non-homologous regions, repeat element proportion in *D. holocanthus *genome increased to 10.6% compared with *T. rubripes *and increased to 9.19% compared with *Te. nigroviridis*. These are obviously higher than the proportion in the sequenced *D. holocanthus *genome (7.94%), and are nearly twice as high as these in the homologous regions (5.16% compared with *T. rubripes *and 4.87% compared with *Te. nigroviridis*). The increasing proportion of repeat elements in the non-homologous region indicates that the rates of repeat element accumulation were inconsistent across different *D. holocanthus *genomic regions. Distribution analysis result showed that transposable elements (e.g., SINEs) have accumulated both in the intergenic regions and introns, which have contributed to genome size variation between *D. holocanthus *and the smooth pufferfish. Previous studies have showed that the profiles of interspersed repeats differed in the smooth pufferfish genomes [13,24]. For example, the most common repeat in the *T. rubripes *genome was LINE-like element Maui, whereas in the *Te. nigroviridis *genome was DNA transposon-Buffy. Unlike in the smooth pufferfish genomes, the most common repeat in the sequenced *D. holocanthus *genome was V-SINE, a kind of non-autonomous retrotransposon. Imai et al. [44] also found that various repetitive elements accounted for genome size variation between *T. rubripes *and *O. latipes *by analyzing chromosome LG22 of *O. latipes *and its corresponding region in *T. rubripes*, which is consistent with our result. However, both the extent and types of repetitive elements which accounted for genome size variation between *T. rubripes *and *O. latipes *are different from our results. More than a half (54%) of genome size variation between *T. rubripes *and *O. latipes *was contributed by various repetitive elements, which is much higher than that between pufferfish in our analysis. Instead of interspersed repeats differently accumulating, genome size variation mainly resulted from different accumulation of unclassified low copy repeats between *T. rubripes *and *O. latipes*. In fact, the inconsistence between our results and Imai et al. [44] might be due to the following reasons. Firstly, different genomic regions were selected for studying. Our synteny analyses showed that selected genomic regions were not overlapped between our study and Imai et al. [44] (see Table 3). Secondly, the most conceivable reason is that results of genome size variation study rely on phylogenetic relationship and divergent time between selected species for comparison. *T. rubripes *diverged from *O. latipes *before 184 million years ago in Imai et al. [44], which is much earlier than the divergence time between *D. holocanthus *and *T. rubripes *(50-70 Mya) in our study (Figure 1). Although inconsistency exists, both our study and Imai et al. [44] supported that different content of repetitive elements accounted for genome size shrinking of smooth pufferfish. Thus, our analysis confirmed the previous DNA renaturation analysis [27] and demonstrated that different content of transposable elements have contributed to the genome size variation in the pufferfish.

Our synteny alignments of the sequenced BAC clones and the genomes of other fish species provided an overview of the genome size variation in the pufferfish. According to the synteny identification, the sequences of the *D. holocanthus *genome could be classified into the following two regions compared with smooth pufferfish genomes: the homologous region, which could be located onto smooth pufferfish genomes; and the non-homologous region, which had no homology with the smooth pufferfish genomes. We inferred that the length of the homologous region in *D. holocanthus *genome was at least 1.37 times longer than that in the *T. rubripes *genome (364 kb vs 265 kb, respectively), and was 1.52 times longer than that in the *Te. nigroviridis *genome (223 kb vs 148 kb, respectively). Therefore, we deduced that the length of the homologous region between *D. holocanthus *and *T. rubripes *was 364 Mb in the *D. holocanthus *genome and was 266 Mb in the *T. rubripes *genome, and that between *D. holocanthus *and *Te. nigroviridis *was 224 Mb in *D. holocanthus *and was 147 Mb in *Te. nigroviridis*. This analysis implied that genome size variation between *D. holocanthus *and smooth pufferfish was partially exhibited as length variations between the homologous regions. Also the lengths of their non-homologous sequences are different; in particular the length of the non-homologous sequence in *D. holocanthus *is longer than that in smooth pufferfish. This is partially explained by the finding that repeat element proportion in *D. holocanthus *genome increased to 10.6% compared with *T. rubripes *and increased to 9.19% compared with *Te. nigroviridis *in the non-homologous regions. Interestingly, *T. rubripes *and *Te. nigroviridis *have such different amounts of sequence both in homologous and non- homologous region compared with *D. holocanthus*. With 2 smooth pufferfish sharing most of the biological traits and equal divergence time with *D. holocanthus*, one explanation for this result is different generation time between *T. rubripes *and *Te. nigroviridis*. The generation time of *Te. nigroviridis *is much shorter (about 1 year) compared with *T. rubripes *(3-4 years), which make they have different evolutionary rate and result in their different genome similarity compared with *D. holocanthus*. A fraction of *D. holocanthus *genomic sequences was not homologous with other fish genomes (e.g., *G. aculeatus *and *O. latipes*) and might be the *D. holocanthus *genome-specific sequence. This part of the *D. holocanthus *genome-specific sequence accounted for 12.1% or approximately 94 Mb of the *D. holocanthus *genome and contributed approximately 21.86% of genome size variation between *D. holocanthus *and the smooth pufferfish. Using *G. aculeatus *and *O. latipes *as reference species, we found that a fraction of the homologous region of the *D. holocanthus *genome (0.1%) might have been lost in the two smooth pufferfish genomes. These results suggested that genome size variation between *D. holocanthus *and the smooth pufferfish was exhibited as the length variation in the homologous region and different levels of the non-homologous sequence accumulation.

The interest in the genome size variation among the pufferfish mainly arises from that the smooth pufferfish have the smallest vertebrate genomes yet measured. If *D. holocanthus *and smooth pufferfish genomes are presently at equilibrium with regard to their size and the tiny genome is a derived character and unique to the smooth pufferfish [23], the difference in genome size between the pufferfish implies that an ancestral equilibrium was disturbed in the smooth tetraodontid lineage following its divergence from the spiny diodontids. The mechanisms of genome size evolution in the pufferfish have been debated in previous studies [13,27]. Aparicio et al. [13] suggested that the rapid deletion of nonfunctional sequences may be the predominant mechanism accounting for the compact genome of *T. rubripes*. Neafsey and Palumbi [27] proposed that a reduction in the rate of large insertions in the smooth puffer, rather than an increase in large deletions, can explain their genomic contraction and difference in size between the smooth and spiny puffer genomes. They also proposed that different transposable element activity might be the main driving force that is responsible for genome size variation between the smooth and spiny puffer genomes. In this study, although the difference amount of repeat elements itself was insufficient to explain the two-fold difference in genome size between *D. holocanthus *and smooth pufferfish, this might be due to that mutations have made many ancient transposable elements unrecognizable. However, we verified that amount and content of transposable elements is different between *D. holocanthus *and the smooth pufferfish. Especially, LINEs with self transposable ability are more prevalent in *D. holocanthus *than that in smooth pufferfish, which means that transposable element activity is different between *D. holocanthus *and the smooth pufferfish. Therefore, our results indicated that the difference of transposable element activity contributed to and genome size variation between *D. holocanthus *and smooth pufferfish and that a reduction in the rate of large insertions caused by transposable elements is responsible to smooth pufferfish genomic contraction as proposed by Neafsey and Palumbi [27].

## Conclusions

To study the genome size variation in the pufferfish, 10 BAC clones of the spiny pufferfish *D. holocanthus *were shotgun sequenced. In total, 776 kb of non-redundant sequences without gaps, constituting 0.1% of the *D. holocanthus *genome, were identified. Sequence analysis showed that *D. holocanthus *has a low gene density and repeat elements rich genome compared with the smooth pufferfish. Further analysis showed that *D. holocanthus *genome is characterized by longer introns and more interspersed repeats compared with the smooth pufferfish genomes. Our analysis also showed that the genome size variation between *D. holocanthus *and smooth pufferfish exhibits as the length variation in the homologous region and the different accumulation of the non-homologous sequence. Our results showed intron size variation was consistent with genome size variation between *D. holocanthus *and smooth pufferfish. We verified that different amount and content of repeat elements, especially the different accumulation of transposable elements, are responsible for the genome size variation between *D. holocanthus *and smooth pufferfish.

## Methods

### BAC clone selection, shotgun sequencing, and assembly

Ten BAC clones of around 100 kb were stochastically selected from a BAC library of the spiny pufferfish *D. holocanthus *and were sequenced using a standard shotgun strategy. Purified *Escherichia coli *genomic DNA-free plasmid DNA was sheared using a HydroShear (Genomic Solutions^®^). The resulting fragments were selected for a size range of 1.5-3.0 kb and extracted by agarose gel electrophoresis (QIAquick Gel Extraction Kit). The selected fragments were randomly inserted into the pUC118 vector (TaKaRa) with T4 ligase (Promega). Sequence reads of randomly selected sub-clones were generated from a single end using with the universal vector M13 primers.

After a sequence redundancy of more than threefold had been generated, a total of 9,286 high-quality reads were edited and assembled using the Phred/Phrap/Consed program package [45-47]. The resultant contigs were then joined into scaffolds based on read-pair associations and order information from the shotgun clones.

Then interspersed repeats were then masked in the scaffolds of each BAC by RepeatMasker (version open-3.1.5) using a repeat library (RepBase14.01) [48]. Masked scaffolds were compared with genomic sequences of *T. rubripes *and *Te. nigroviridis *using the Basic Local Alignment Search Tool (BLAST) algorithm with the default parameters[30]. The BLAST results were first clustered on the basis of colinearity for further screening. Manual inspection readily identified spurious hits which are mainly resulted from repetitive sequences and are not identified by RepeatMasker, and poor hits (identical base pairs < 100) arising from lineage divergence. After removing these spurious hits, we ordered and oriented as many super-scaffolds as possible within the same BAC clone by comparison with the genomes of *T. rubripes *and *Te. nigroviridis*, and the gaps were represented with 100 Ns.

### Sequence annotation and data analyses

An integrative gene annotation process was performed mainly based on the Ensembl pipeline of gene annotation with some modifications [49]. Two *ab initio *gene prediction programs, FgenesH 2.6 [28] and GENSCAN 1.0 [29] were firstly adopted to make *de novo *gene prediction. TBLASTN [30] combined with GeneWise [31] analyses were then used for further gene predication. TBLASTN allows similarity searches against peptides database, and it was used with an E value of 1 × 10^-6 ^as the cutoff for identifying potential orthologous genes in the *T. rubripes *genome and the *Te. nigroviridis *genome in the Ensembl protein dataset (release 52). In total, 144 candidate peptides were identified in the *T. rubripes *genome and 67 in the *Te. nigroviridis *genome, and those showing more than 50% of identity and 70% of peptide length were maintained. This part of peptides was subsequently subjected to GeneWise analysis against the *D. holocanthus *genome sequence, taking into account splice sites and frameshifts. The best predictions were considered as overlapping results with above annotation steps and were integrated together to yield a summary of the annotated genes. This set of predictions was then manually filtered for redundancy resulted from alternative splicing or paralogous genes. The final predicted outputs were used as the input for the BLASTP [30] searches against the non-redundant GenBank protein database to assign their possible identity.

Genomic synteny were identified using BLASTN [30] searches between the sequenced *D. holocanthus *genome and the genomes of *T. rubripes*, *Te. nigroviridis*, *G. aculeatus*, and *O. latipes*. The criteria used to filter the BLASTN results were as follows: more than 50% identity and 70% of the query sequence length were maintained in the hit regions; more than two hits occurred between the query and the subject sequence, or if only one hit occurred, the hit length is longer than 300 bp. Homologous sequences were extracted using Perl scripts. The results were manually inspected and the synteny maps were visualized using the Artemis Comparison Tool (ACT) [50].

Repetitive element identification in *D. holocanthus *genome sequences was accomplished through RepeatMasker (version open-3.1.5), CENSOR [51], and BLAST similarity searches against known elements in REPBASE (version 14.01) [48] and GenBank. Repetitive elements were also identified using RepeatMasker (version open-3.1.5) in the *T. rubripes *and *Te. nigroviridis *genome with their genome sequences from the Ensembl DNA dataset (release 52). The repeat elements in the homologous regions between the spiny pufferfish *D. holocanthus *and the smooth pufferfish were identified, according to the genomic synteny analyses.

## Authors' contributions

SH and BG conceived the project. BG and XG performed the experiments. BG and MZ analyzed the data. BG and SH wrote the paper. All authors have read and approved the final manuscript.

## Supplementary Material

Additional file 1 **Synteny maps**. The figures show the synteny relationships between the BAC clone sequences of *D. holocanthus *and the smooth pufferfish *T. rubripes *and *Te*. *nigroviridis *genome. Click here for file
