# Supplementary material for: Genome size evolution in pufferfish: an insight from BAC clone-based Diodon holocanthus genome sequencing
Source: BMC Genomics. 2010 Jun 23;11:396. doi: 10.1186/1471-2164-11-396 (PMC2996927; doi:10.1186/1471-2164-11-396)
Supplement: Additional file 1 — Synteny maps. The figures show the synteny relationships between the BAC clone sequences of D. holocanthus and the smooth pufferfish T. rubripes and Te. nigroviridis genome. [file 1471-2164-11-396-S1.PPT]

## Slide 1
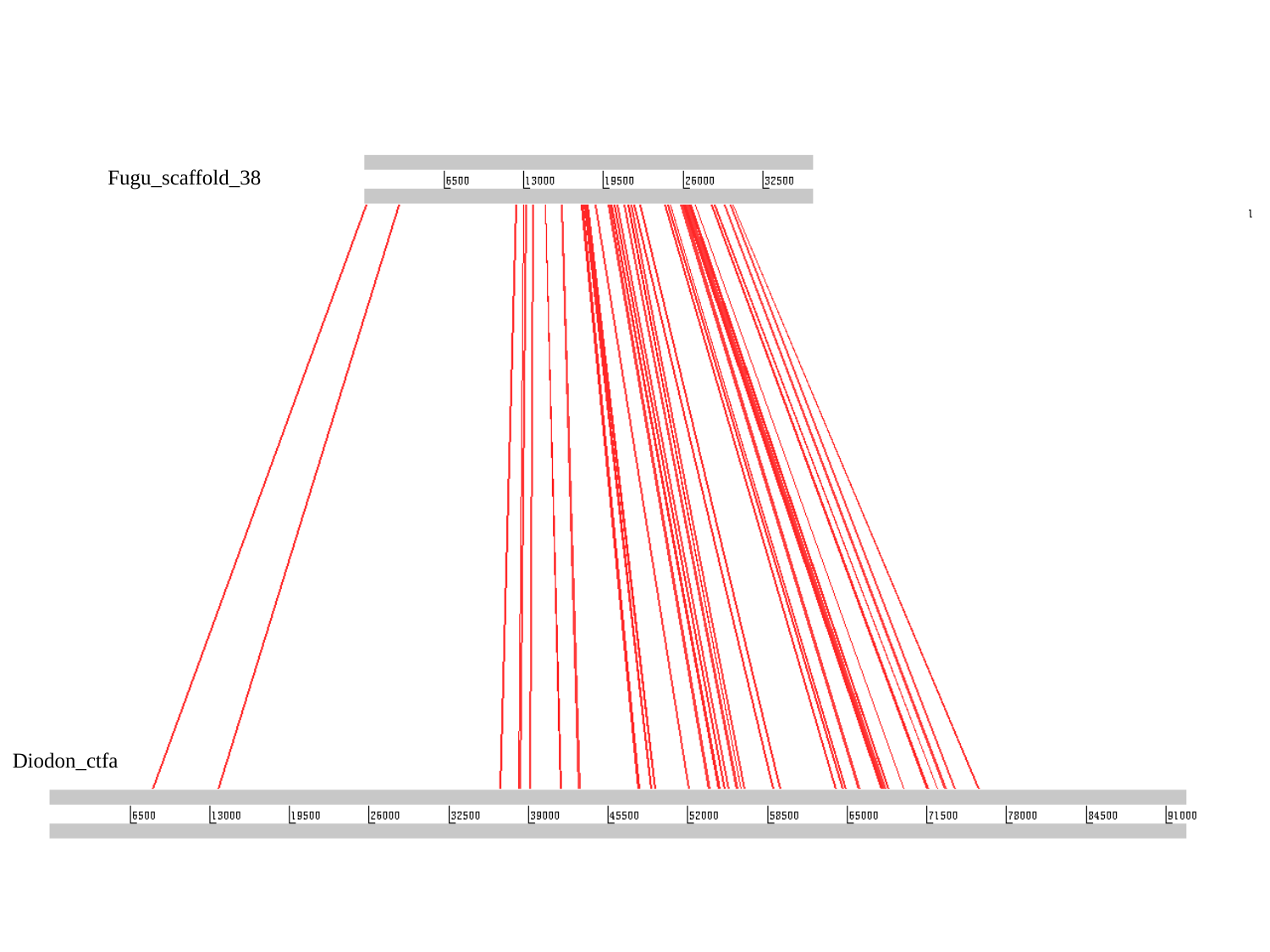

Fugu_scaffold_38
Diodon_ctfa

## Slide 2
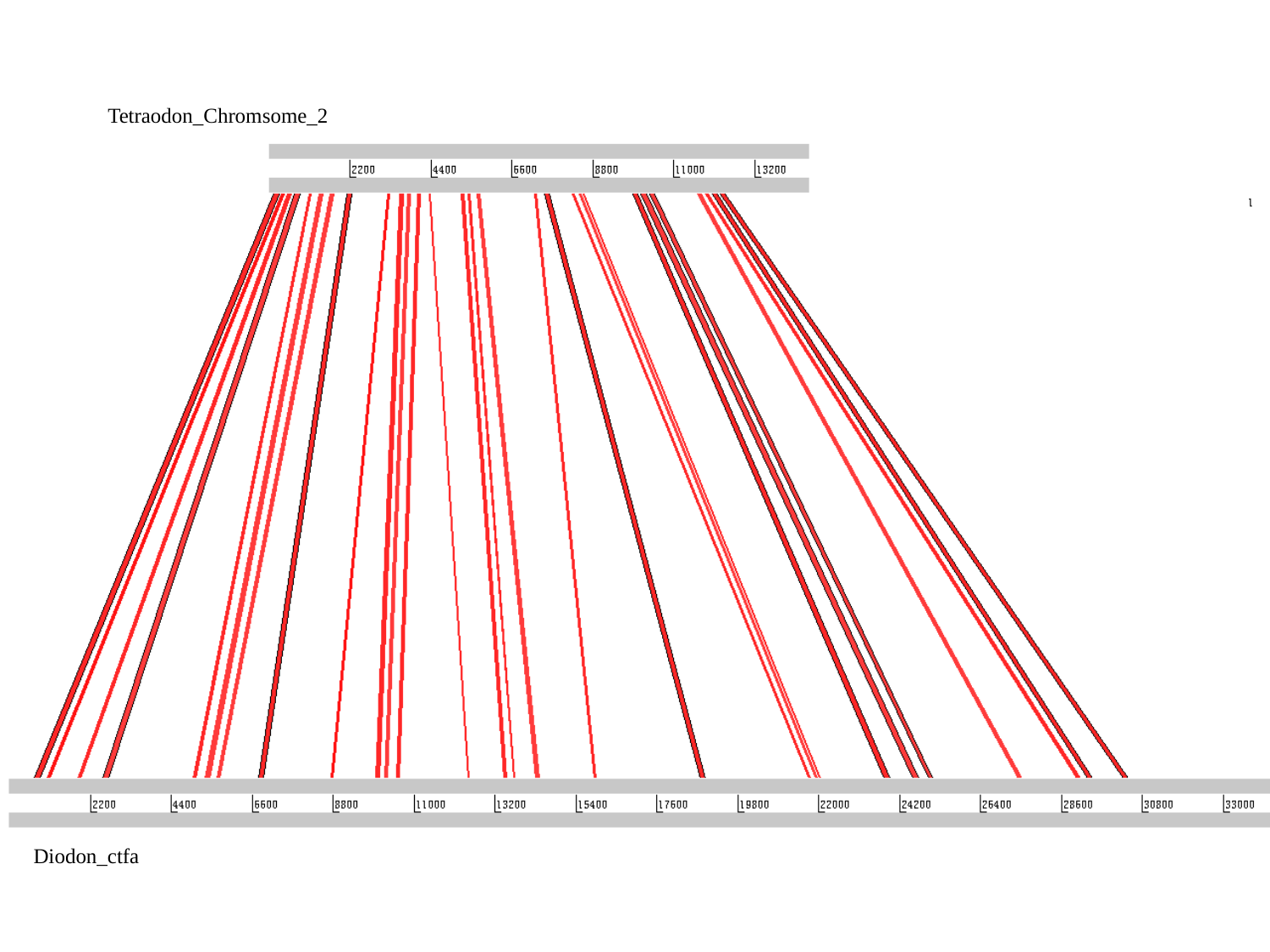

Tetraodon_Chromsome_2
Diodon_ctfa

## Slide 3
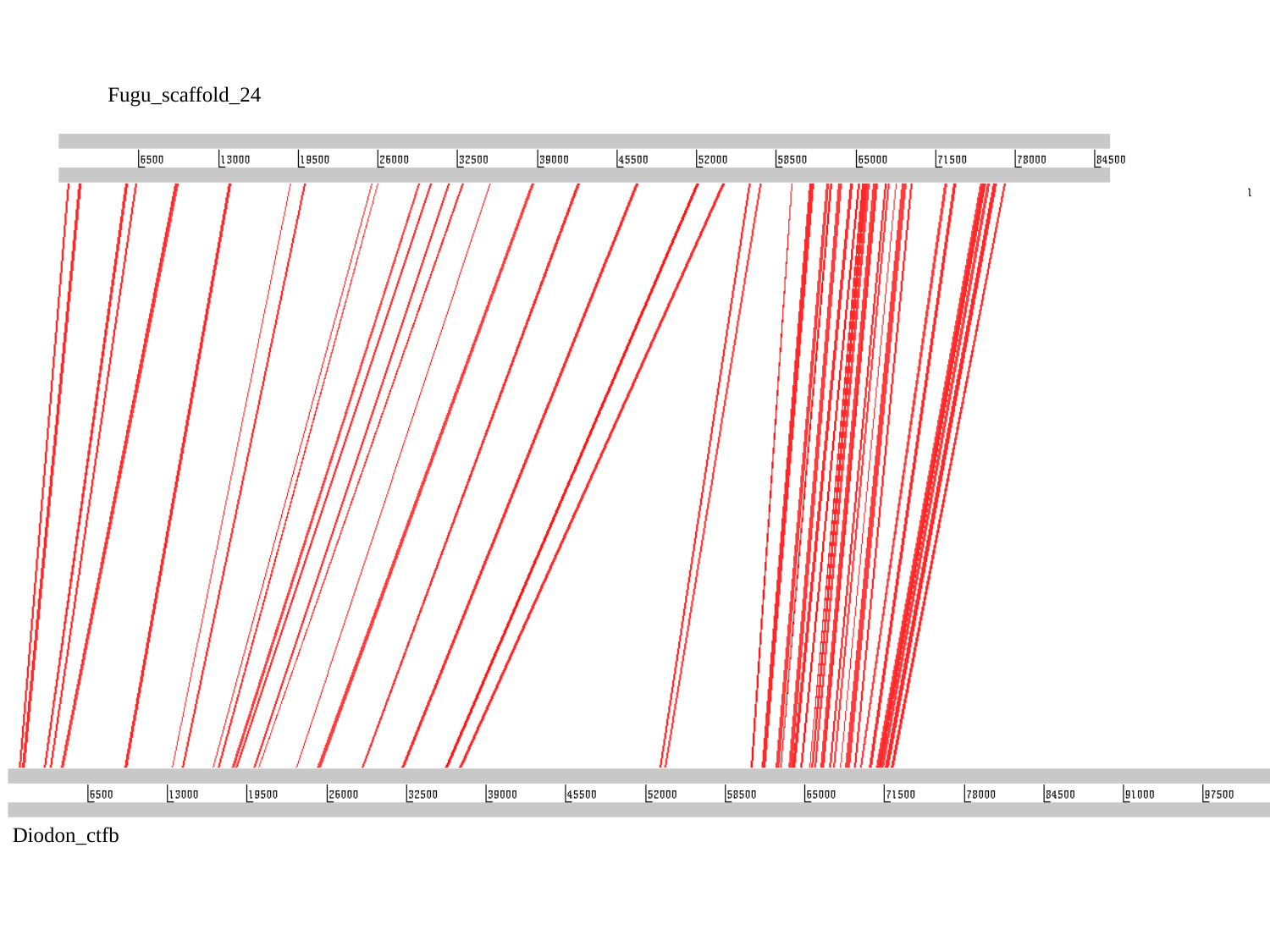

Fugu_scaffold_24
Diodon_ctfb

## Slide 4
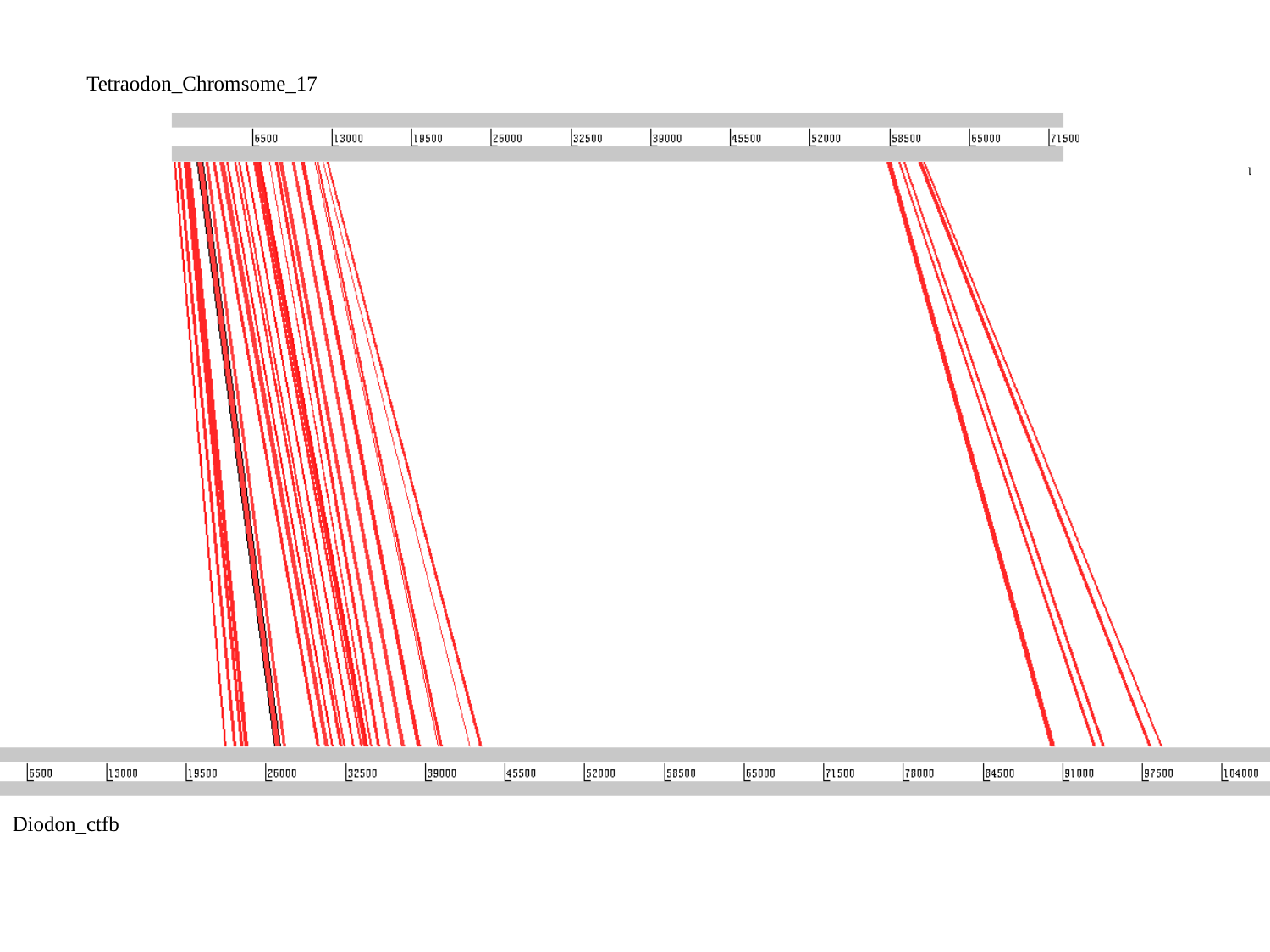

Tetraodon_Chromsome_17
Diodon_ctfb

## Slide 5
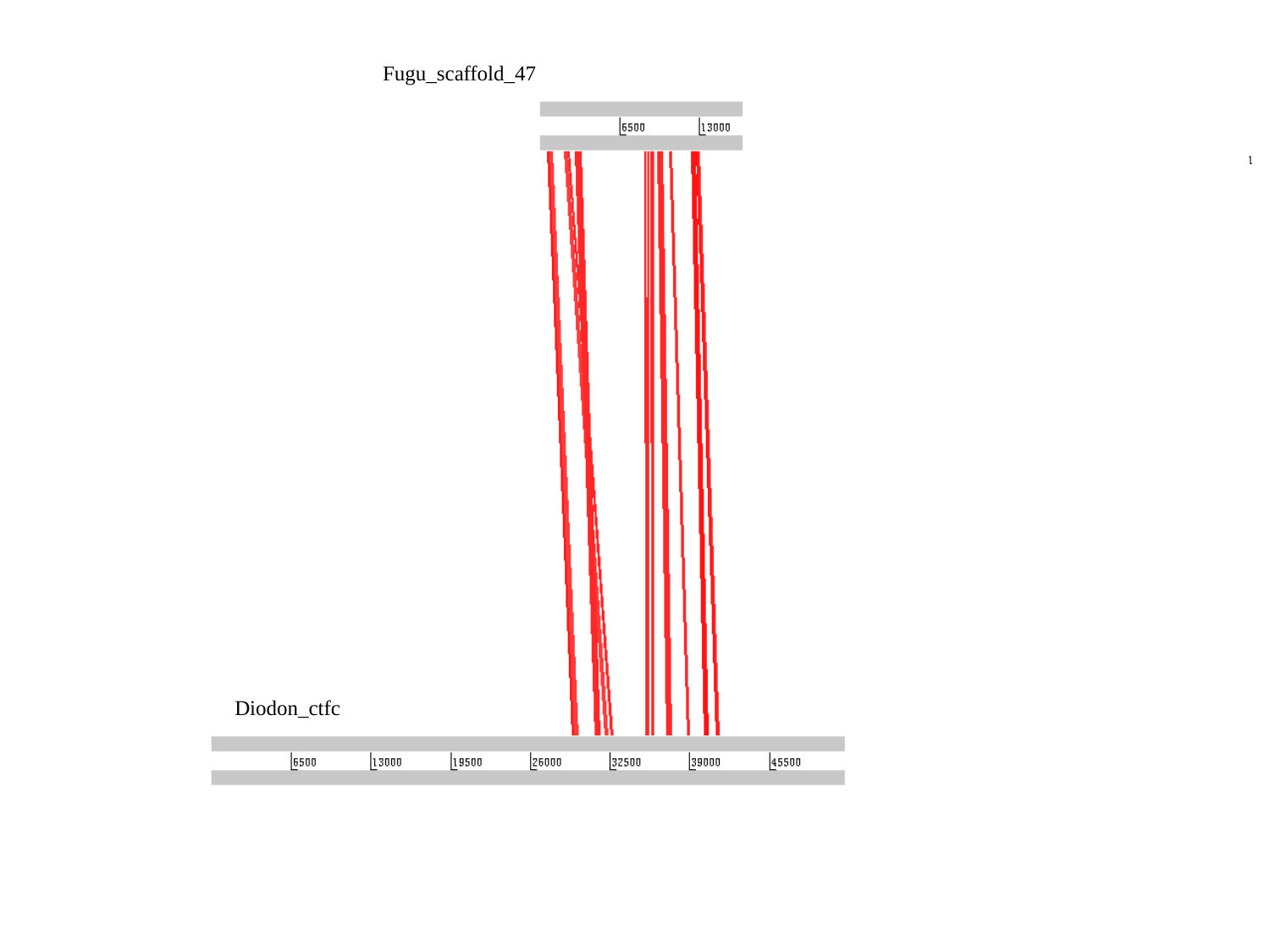

Fugu_scaffold_47
Diodon_ctfc

## Slide 6
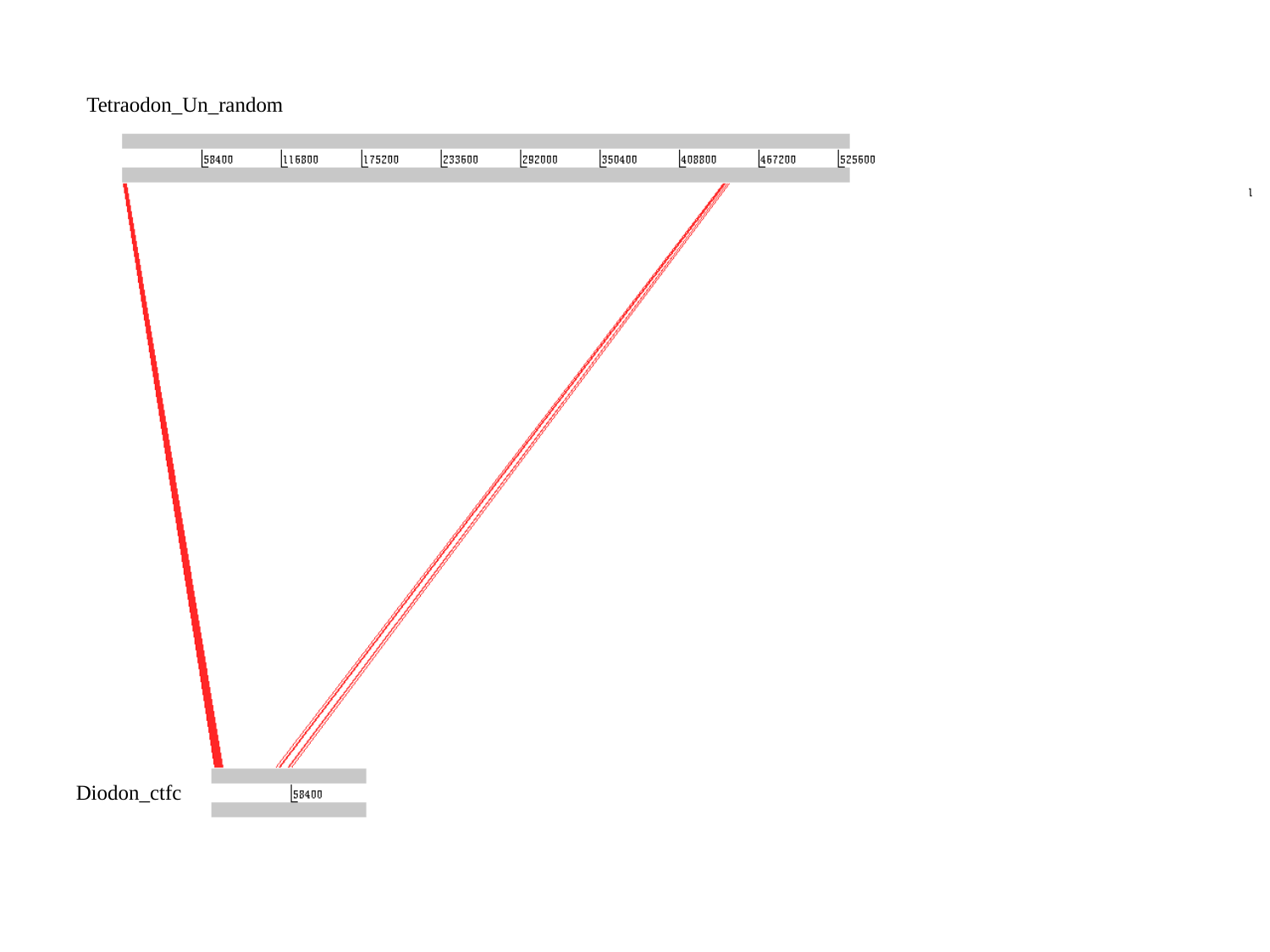

Tetraodon_Un_random
Diodon_ctfc

## Slide 7
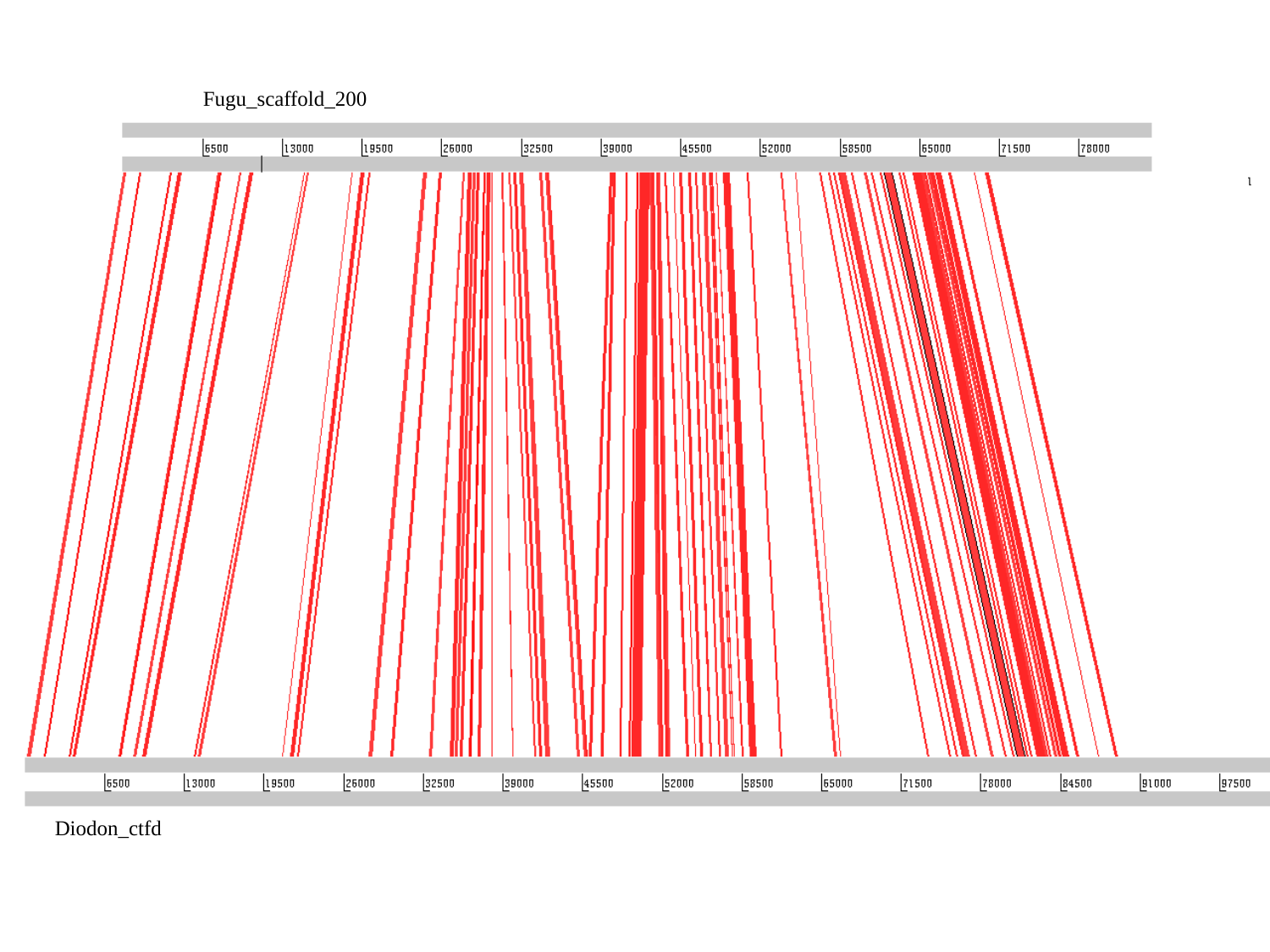

Fugu_scaffold_200
Diodon_ctfd

## Slide 8
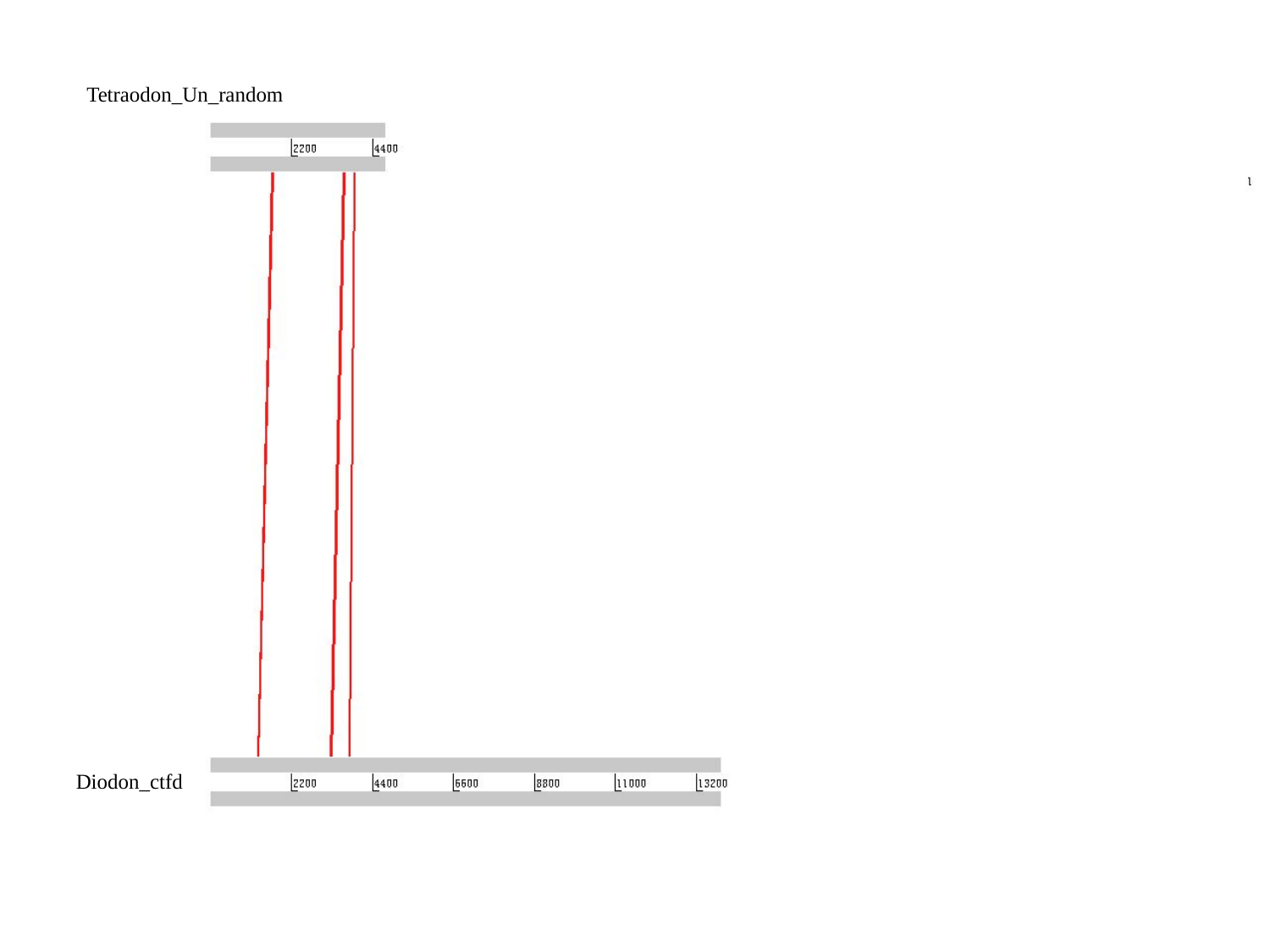

Tetraodon_Un_random
Diodon_ctfd

## Slide 9
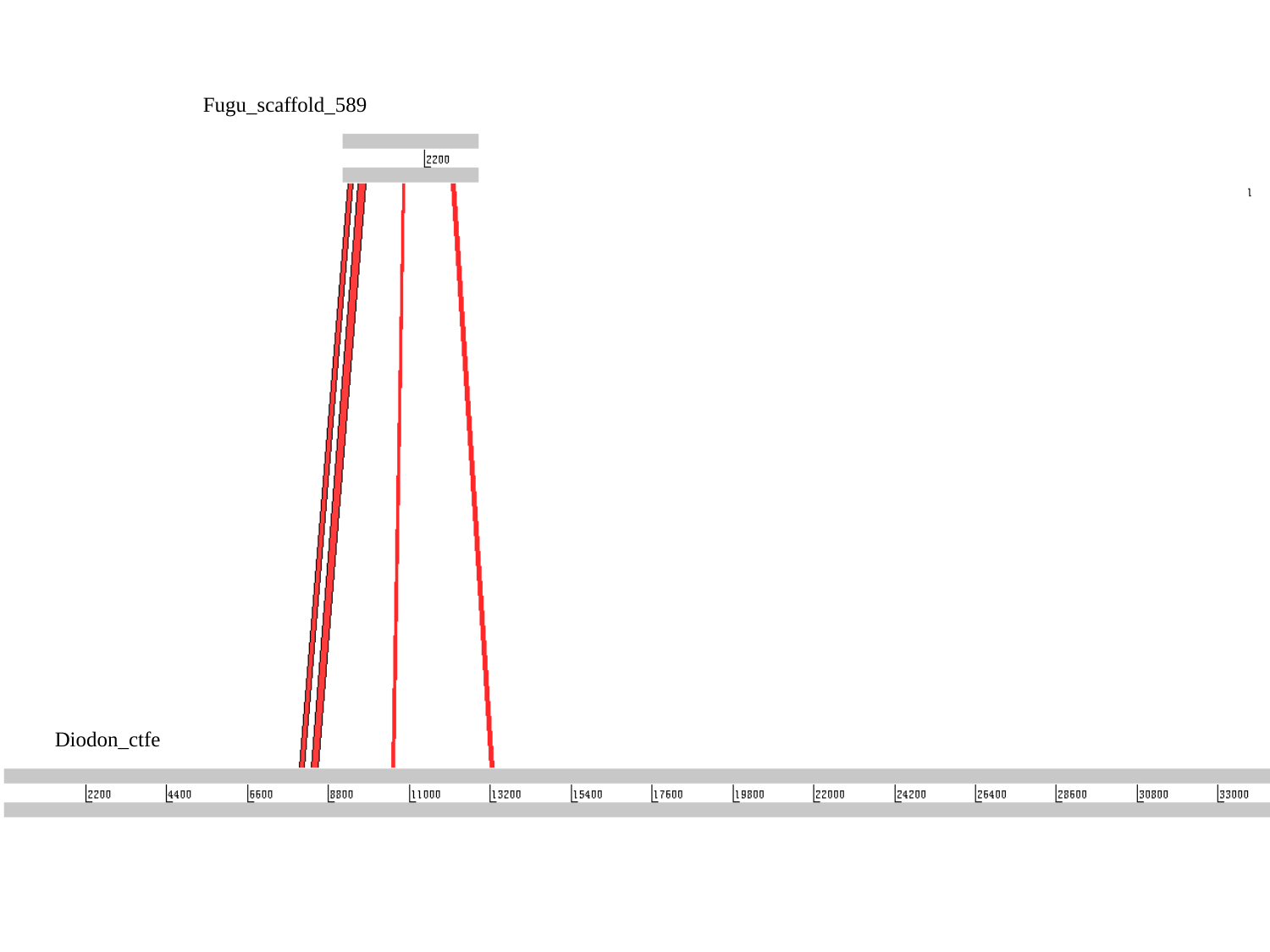

Fugu_scaffold_589
Diodon_ctfe

## Slide 10
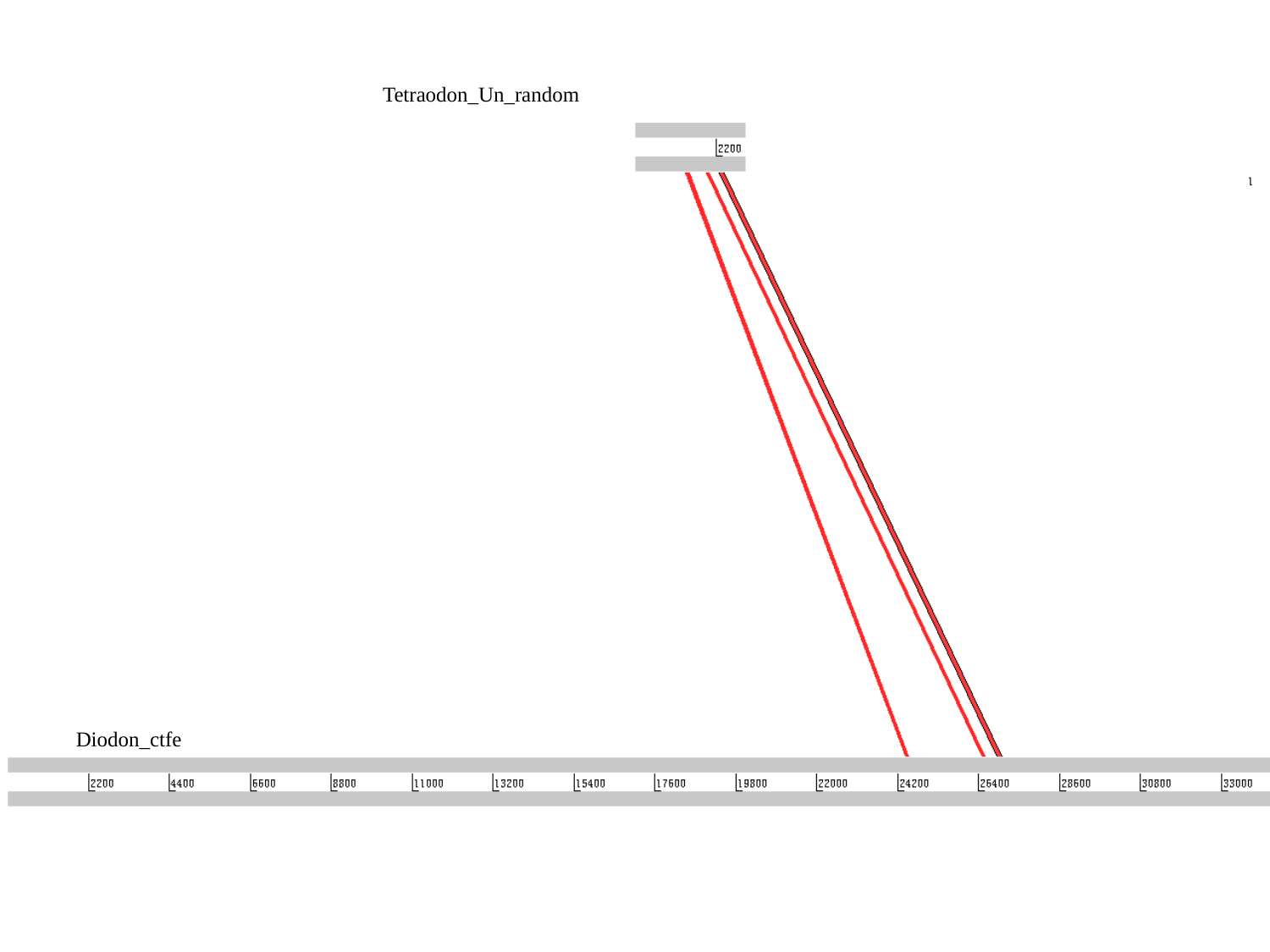

Tetraodon_Un_random
Diodon_ctfe

## Slide 11
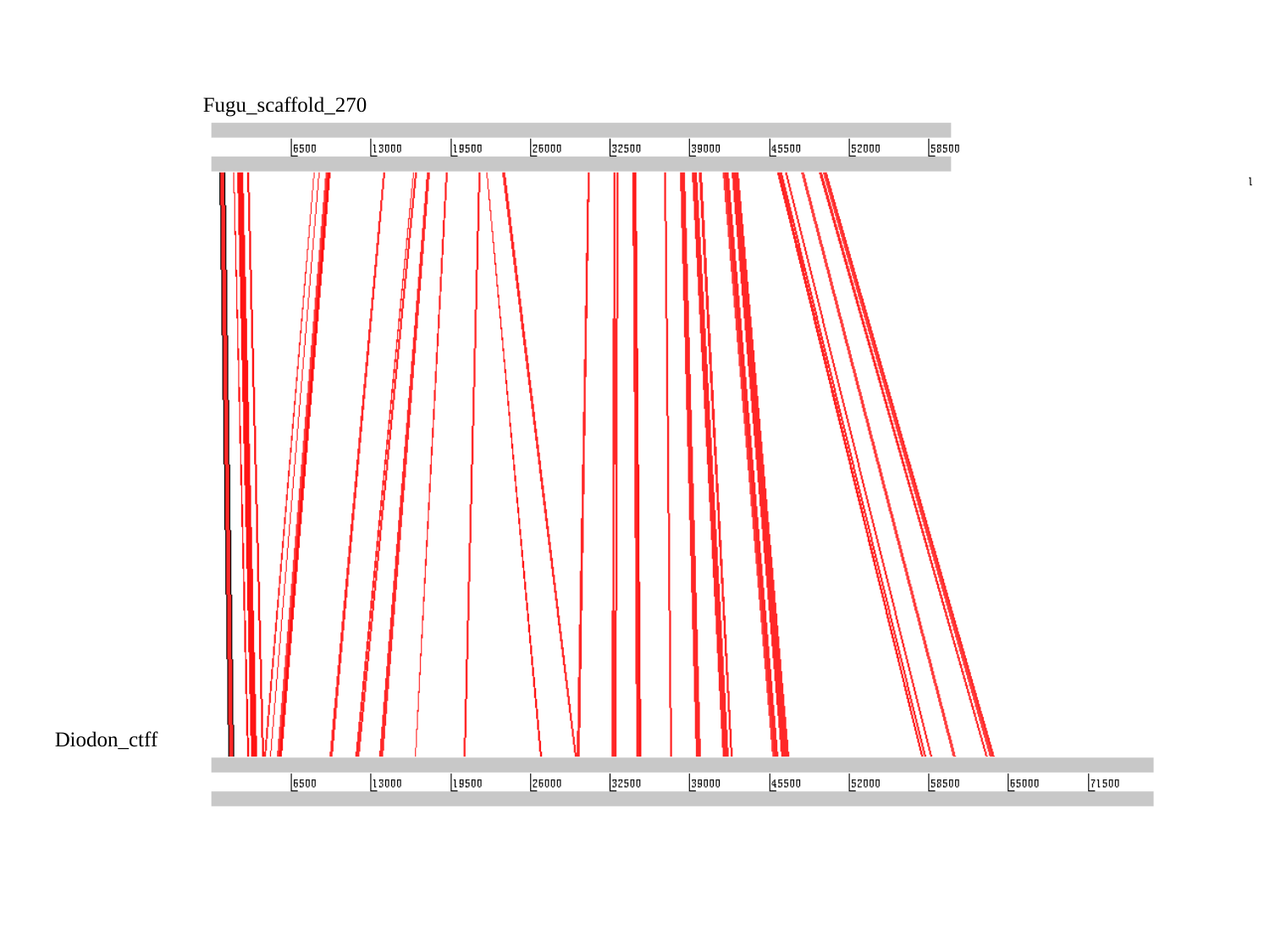

Fugu_scaffold_270
Diodon_ctff

## Slide 12
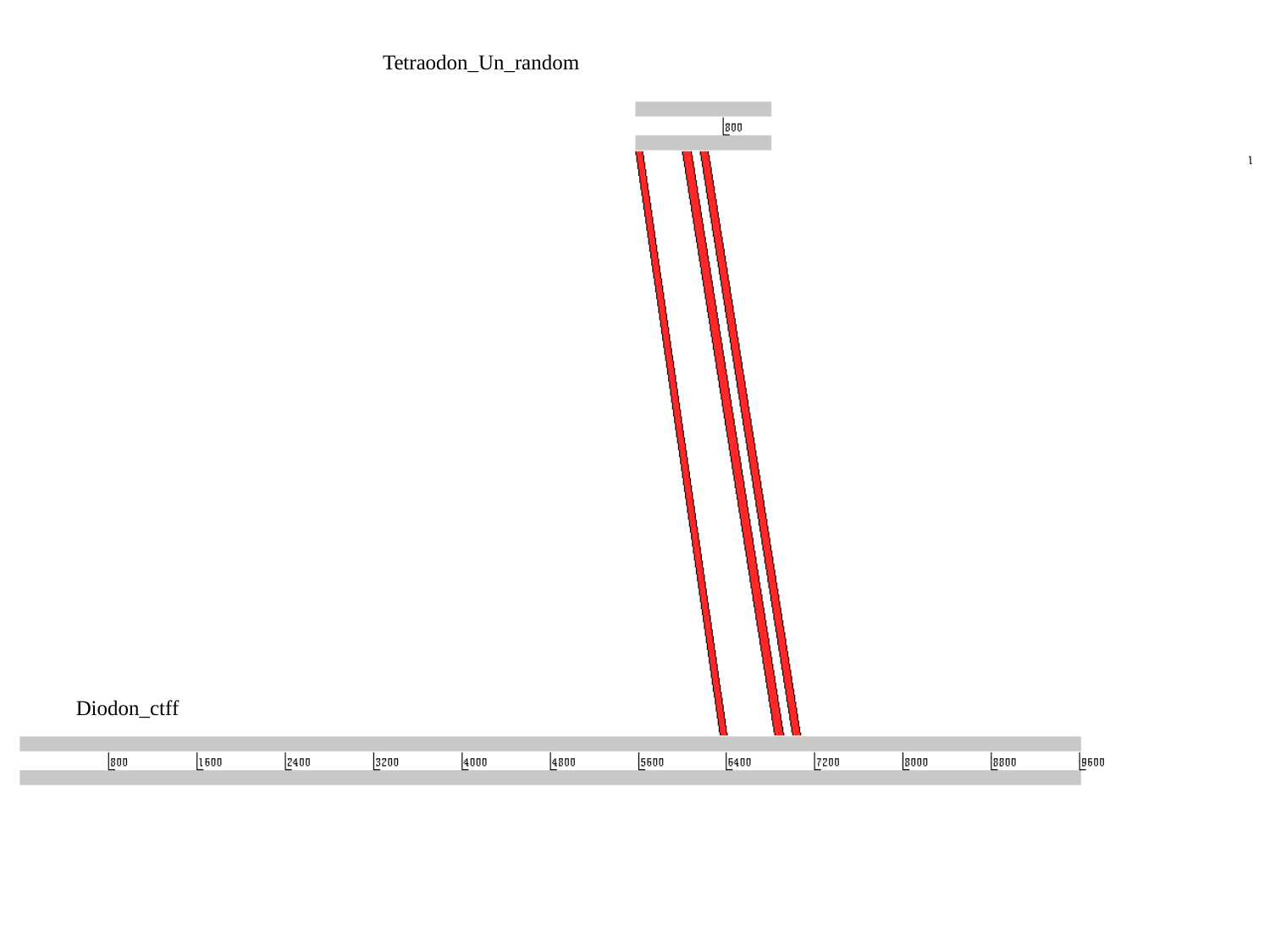

Tetraodon_Un_random
Diodon_ctff

## Slide 13
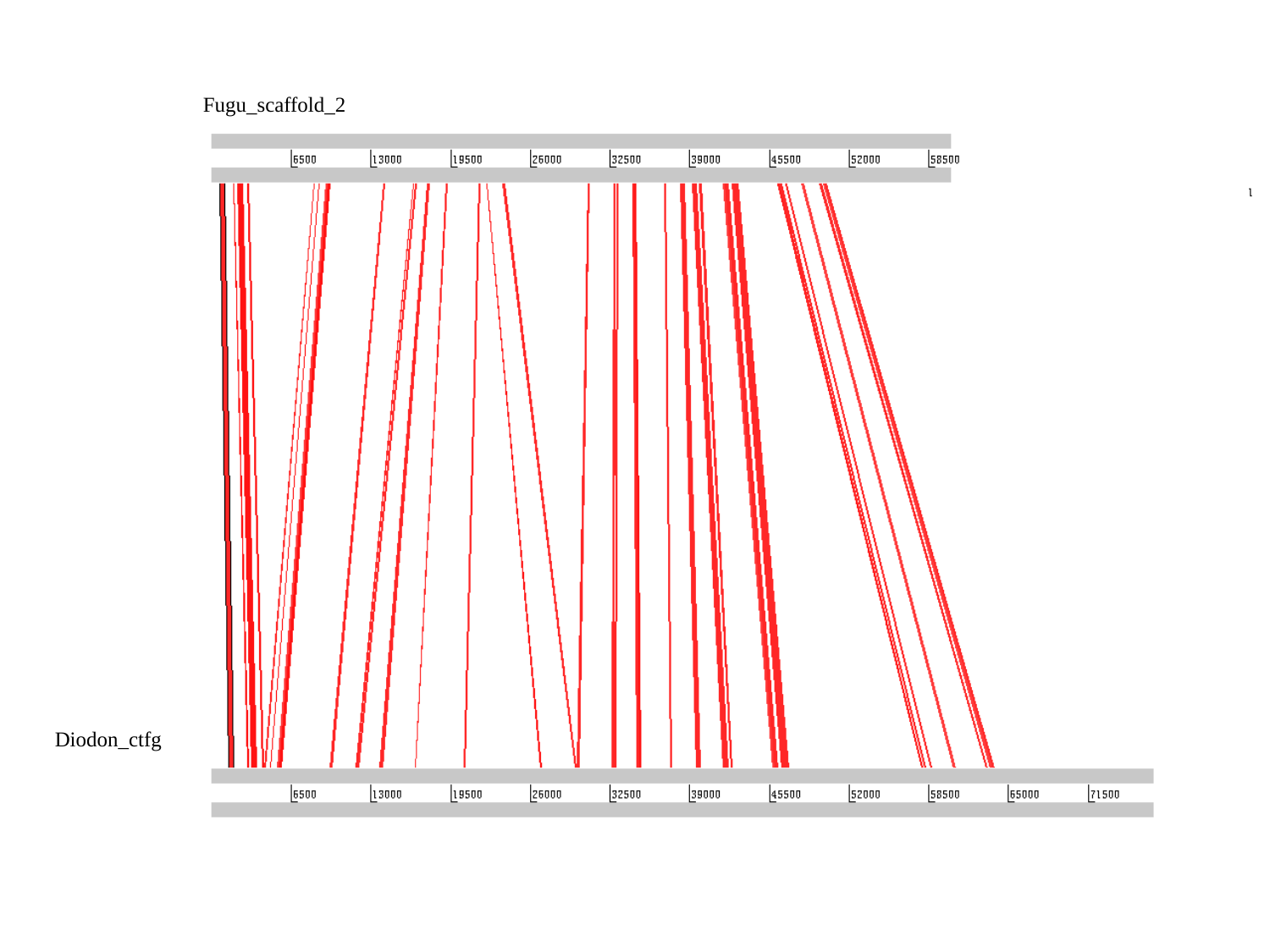

Fugu_scaffold_2
Diodon_ctfg

## Slide 14
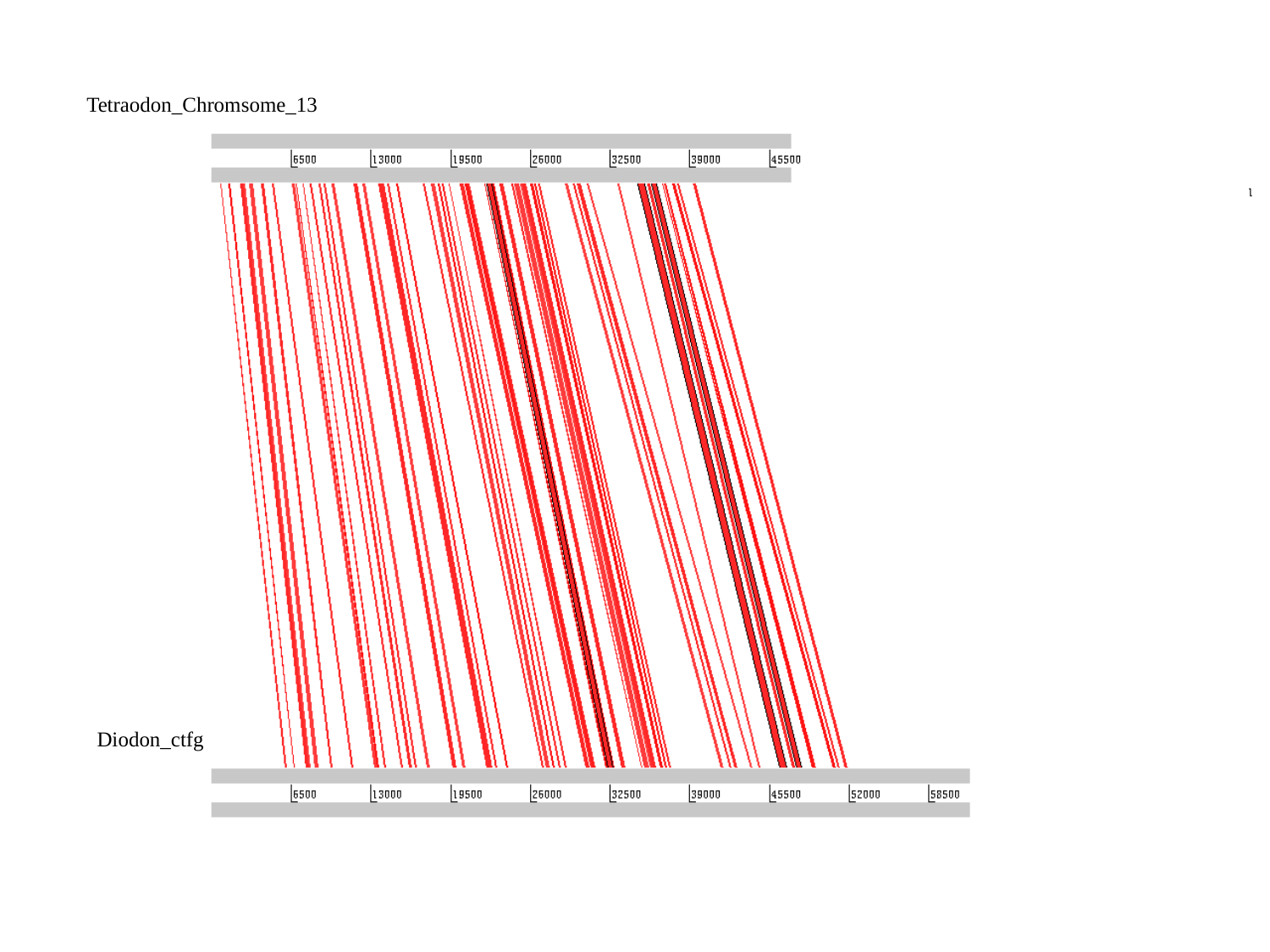

Tetraodon_Chromsome_13
Diodon_ctfg

## Slide 15
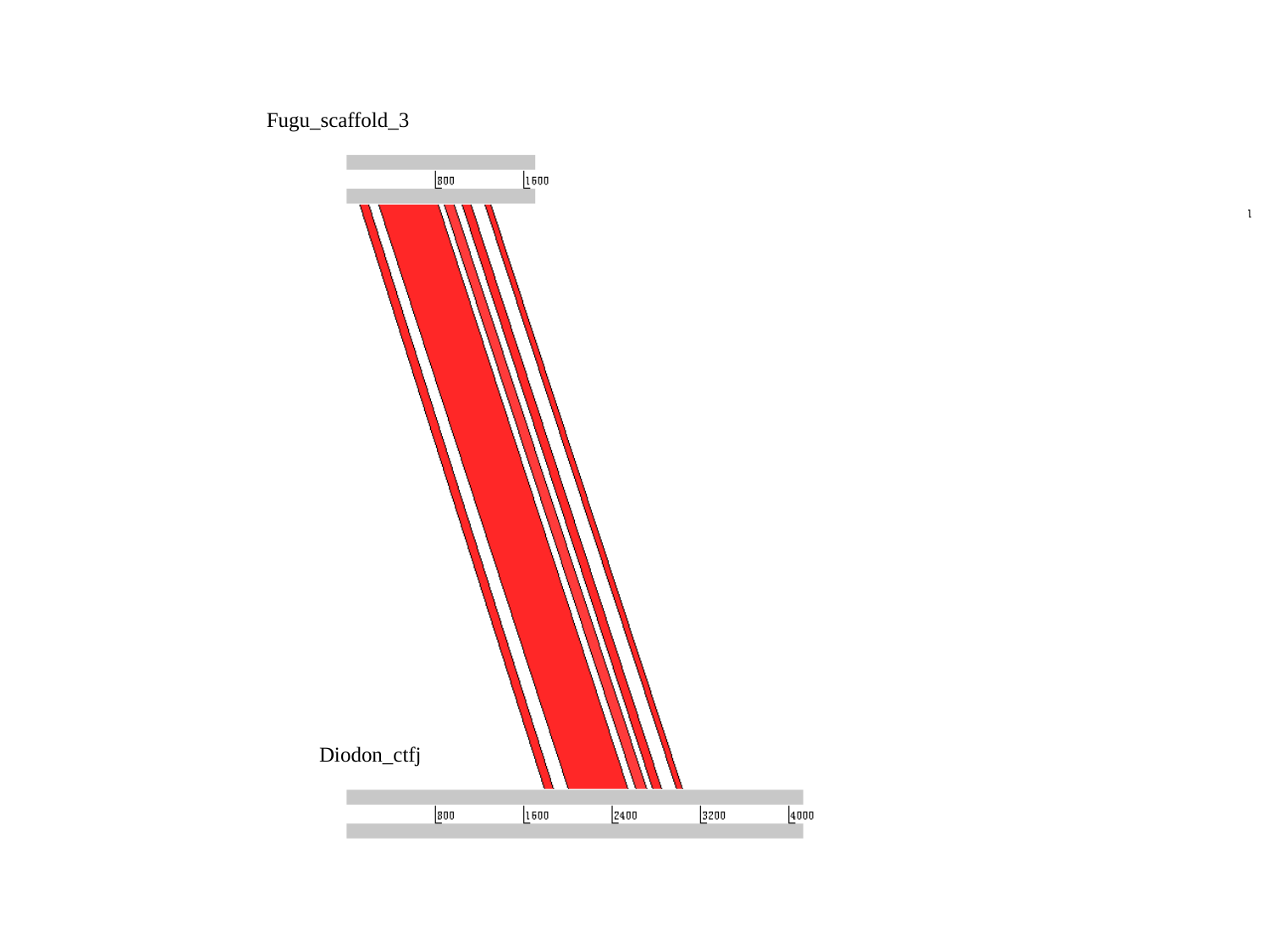

Fugu_scaffold_3
Diodon_ctfj

## Slide 16
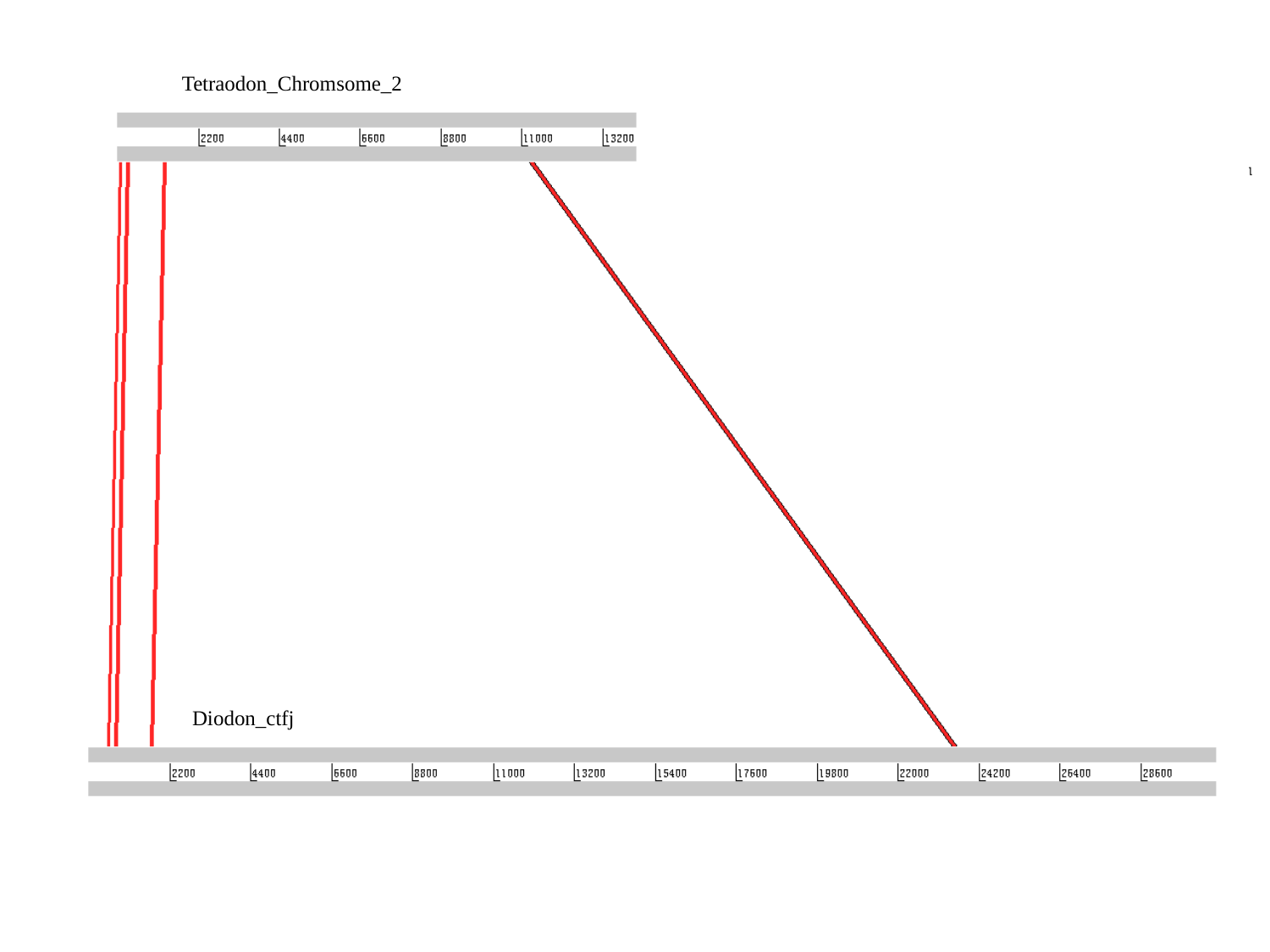

Tetraodon_Chromsome_2
Diodon_ctfj

## Slide 17
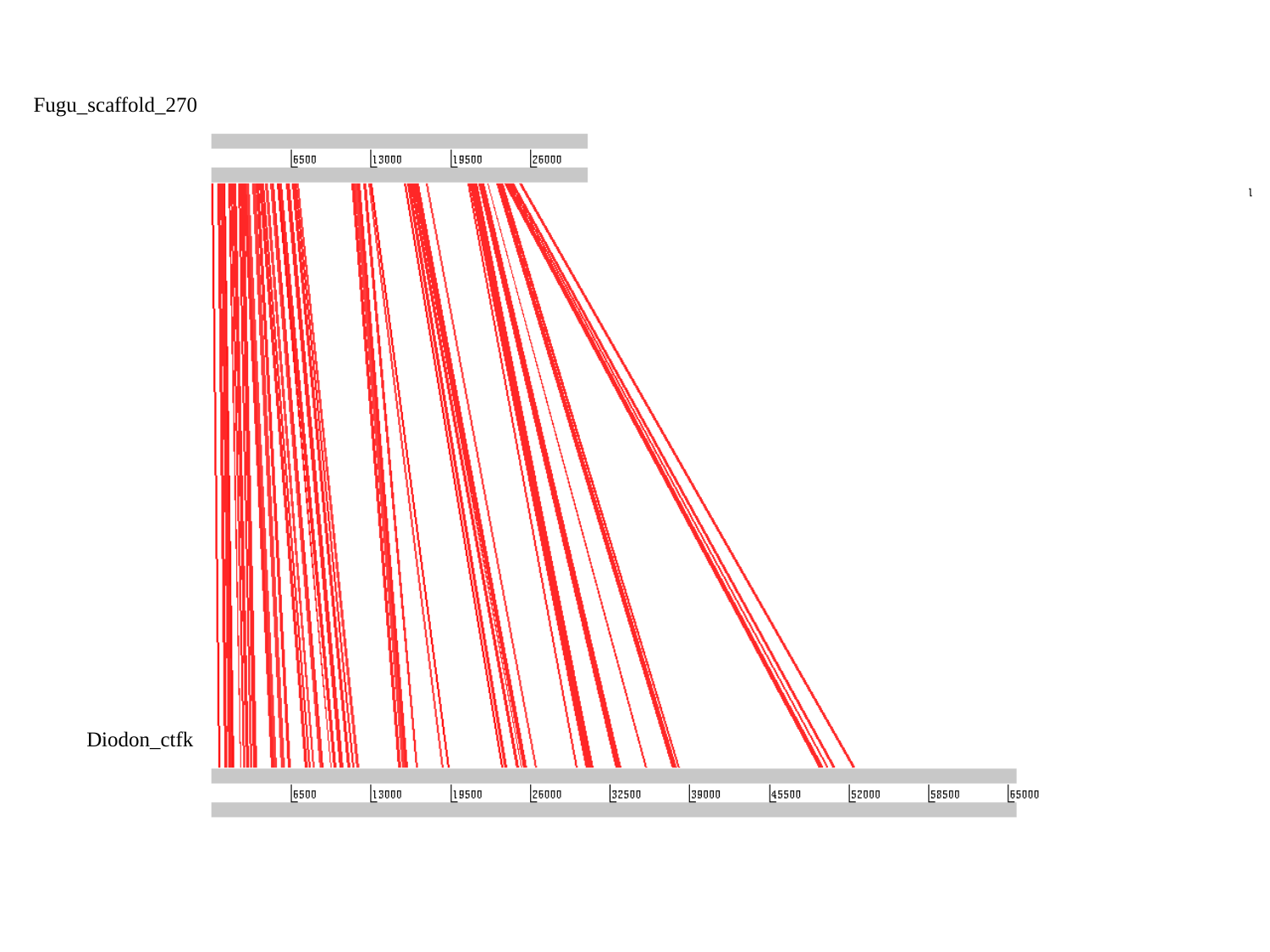

Fugu_scaffold_270
Diodon_ctfk

## Slide 18
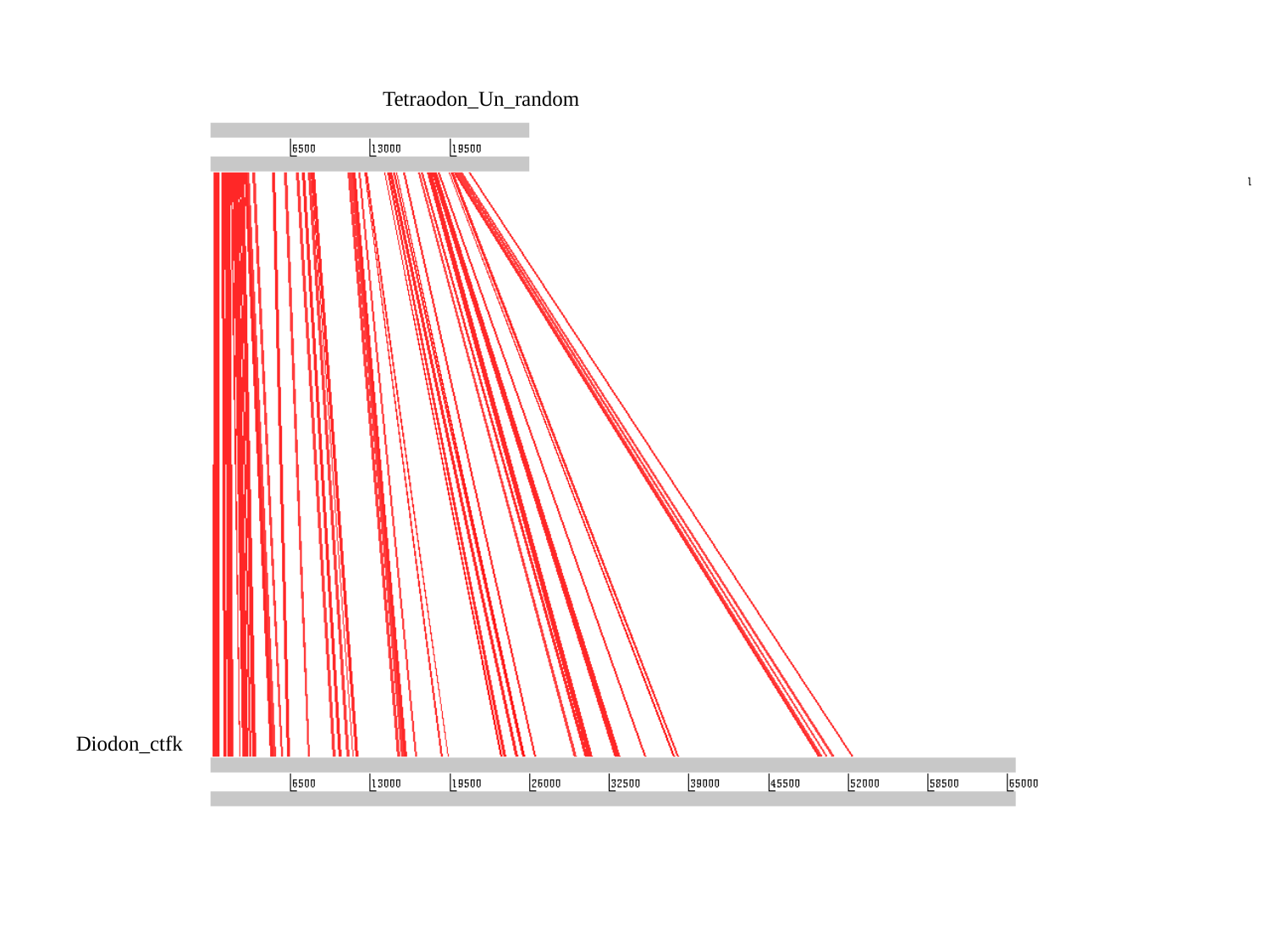

Tetraodon_Un_random
Diodon_ctfk
